# Target-Oriented Synthesis of Marine Coelenterazine Derivatives with Anticancer Activity by Applying the Heavy-Atom Effect

**DOI:** 10.3390/biomedicines9091199

**Published:** 2021-09-11

**Authors:** Carla M. Magalhães, Patricia González-Berdullas, Diana Duarte, Ana Salomé Correia, José E. Rodríguez-Borges, Nuno Vale, Joaquim C. G. Esteves da Silva, Luís Pinto da Silva

**Affiliations:** 1 Chemistry Research Unit (CIQUP), Faculty of Sciences, University of Porto, Rua do Campo Alegre 687, 4169-007 Porto, Portugal; up201201533@edu.fc.up.pt (C.M.M.); patricia.berdullas@fc.up.pt (P.G.-B.); jcsilva@fc.up.pt (J.C.G.E.d.S.); 2 OncoPharma Research Group, Center for Health Technology and Services Research (CINTESIS), Rua Doutor Plácido da Costa, 4200-450 Porto, Portugal; dianaduarte29@gmail.com (D.D.); anncorr07@gmail.com (A.S.C.); nunovale@med.up.pt (N.V.); 3 Department of Community Medicine, Health Information and Decision (MEDCIDS), Faculty of Medicine, University of Porto, Alameda Professor Hernâni Monteiro, 4200-319 Porto, Portugal; 4 LAQV/REQUIMTE, Department of Chemistry and Biochemistry, Faculty of Sciences, University of Porto, Rua do Campo Alegre 697, 4169-007 Porto, Portugal; jrborges@fc.up.pt; 5 LACOMEPHI, GreenUPorto, Department of Geosciences, Environment and Territorial Planning, Faculty of Sciences, University of Porto, Rua do Campo Alegre 697, 4169-007 Porto, Portugal

**Keywords:** photodynamic therapy, cancer, coelenterazine, chemiluminescence, heavy-atom effect, triplet chemiexcitation, self-activating photosensitizers

## Abstract

Photodynamic therapy (PDT) is an anticancer therapeutic modality with remarkable advantages over more conventional approaches. However, PDT is greatly limited by its dependence on external light sources. Given this, PDT would benefit from new systems capable of a light-free and intracellular photodynamic effect. Herein, we evaluated the heavy-atom effect as a strategy to provide anticancer activity to derivatives of coelenterazine, a chemiluminescent single-molecule widespread in marine organisms. Our results indicate that the use of the heavy-atom effect allows these molecules to generate readily available triplet states in a chemiluminescent reaction triggered by a cancer marker. Cytotoxicity assays in different cancer cell lines showed a heavy-atom-dependent anticancer activity, which increased in the substituent order of hydroxyl < chlorine < bromine. Furthermore, it was found that the magnitude of this anticancer activity is also dependent on the tumor type, being more relevant toward breast and prostate cancer. The compounds also showed moderate activity toward neuroblastoma, while showing limited activity toward colon cancer. In conclusion, the present results indicate that the application of the heavy-atom effect to marine coelenterazine could be a promising approach for the future development of new and optimized self-activating and tumor-selective sensitizers for light-free PDT.

## 1. Introduction

Photodynamic therapy (PDT) is a clinically approved cancer treatment with great potential due to its minimally invasive nature, fewer side effects, and fast healing rate of healthy tissues [1,2]. PDT consists of the irradiation of a tumor site, in which a photosensitizer is accumulated, with light of a specific wavelength. Upon photoexcitation, the photosensitizer will be excited from its ground state (*S*_0_) to its lowest singlet excited state *(S*_1_) and will undergo intersystem crossing (ISC) to triplet states (*T*_1_), now capable of sensitizing the highly cytotoxic singlet oxygen after reacting with triplet oxygen [1,3]. Unfortunately, the low penetration of light into biologic tissues limits this therapy to the treatment of superficial tumors or tumors on the outer lining of internal organs and cavities [4,5]. Furthermore, PDT is also unable to treat metastatic tumors due to these localization-based limitations [5]. Given this, the development of novel tumor-selective photosensitizers capable of intracellular activation without an external light source is essential for eliminating PDT restrictions regarding tumor size and localization [4,6].

**Scheme 1:**
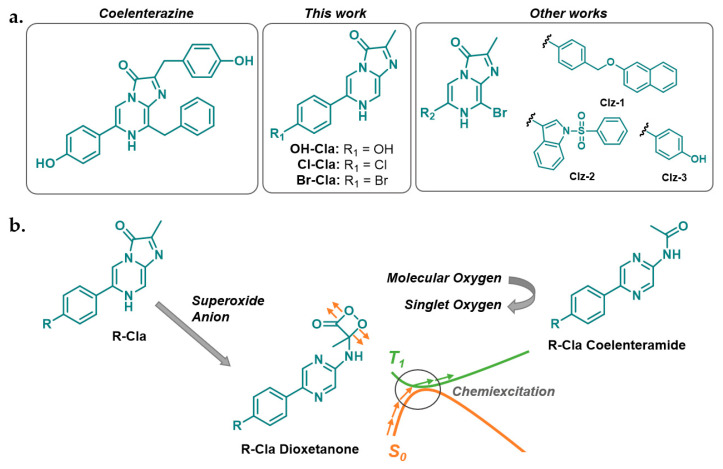
Chemical structures of coelenterazine and derivatives (**a**). Schematic representation of the proposed tumor-selective and self-activating photodynamic therapy based on the chemiluminescent reaction of R-Cla (**b**).

Theoretically, triplet excited states can be generated in a light-free and tumor-selective way, by using the chemiluminescent reaction of marine coelenterazine (Clz) (Scheme 1a) [6,7]. Chemiluminescence consists of the conversion of thermal energy into excitation energy due to a chemical reaction, without an excitation source [6]. For Clz, specifically, the chemiluminescent reaction consists of its oxidation into an unstable cyclic peroxide intermediate (dioxetanone), which rapidly decomposes into the light-emitter, coelenteramide. The chemiluminescence of Clz has the advantage of being triggered solely by superoxide anion, a reactive oxygen species (ROS) typically overexpressed in cancer cells, without requiring any catalyst/cofactor (Scheme 1b) [8]. The key step for chemiexcitation is the decomposition of dioxetanone, during which *S*_0_ becomes degenerated with both *T*_1_ and *S*_1_ states [9]. However, for Clz, the *S*_0_ → *T*_1_ ISC pathway is generally thought of as inefficient, as this system is only known for its efficient generation of singlet excited states [10,11].

Enhancing the *S*_0_ → *T*_1_ ISC pathway can then enable the use of the chemiluminescent reaction of Clz as a self-excitation mechanism to directly generate triplet states able to sensitize singlet oxygen, thus leading to a light-free intracellular reaction exclusively triggered by a cancer marker (superoxide anion). One of the most effective strategies to enhance the efficiency of ISC pathways is through the heavy-atom effect (e.g., introduction of halogen atoms) [3]. Accordingly, we recently synthesized 6-(4-bromophenyl)-2-methylimidazo [1, 2−*a*] pyrazin-3(7*H*)-one (Br-Cla) (Scheme 1a), a brominated Clz derivative in which the hydroxyl of the phenol group was substituted by bromine, and the *p*-cresol and benzyl moieties of Clz were replaced by a methyl group and a hydrogen atom, respectively [12]. Br-Cla showed superoxide-triggered singlet oxygen sensitization and presented anticancer activity toward prostate and breast cancer without inducing toxicity toward noncancer cells [12,13]. Given the promising results and the interesting profile of tumor-selectivity, we continued our research focus and reported the synthesis of three other brominated Clz derivatives (Clz-1, Clz-2, and Clz-3) (Scheme 1a) [13]. These novel compounds showed potential for superoxide-induced generation of triplet states via a CL reaction, while also presenting relevant anticancer activity toward breast and prostate cancer.

Thus, studies with Br-Cla and the remaining brominated Clz derivatives (Scheme 1) provided results indicating that these compounds possess significant potential as prototypical light-free and self-activating single-molecule photosensitizers. However, as all developed derivatives bore a bromine heteroatom, it is not clear if the resulting anticancer activity results from the heavy-atom effect on the CL reaction or if it results from an intrinsic activity related to the imidazopyrazinone core (common to all). Such information is essential to understand if we are indeed in the presence of prototypical light-free and self-activating photosensitizers or in the presence of new potential chemotherapeutic drugs with tumor-selectivity characteristics [12,13].

To further understand the true potential of these Clz derivatives as novel anticancer compounds, it is also important to know how they behave toward different cancer types and assess their spectrum of application. So far, our knowledge in this regard is limited to their application to prostate and breast cancer [12,13].

Herein, we aimed to clarify these topics by employing a target-oriented approach. Specifically, we report the synthesis of three Cla derivatives (OH-, Cl-, and Br-substituted Cla, Scheme 1a), with the rationale that the substitution with halogen atoms of increasing size should enhance the heavy-atom effect into the studied system. If successful, this should increase the ISC rate of the *S*_0_ → *T*_1_ pathway present in the chemiluminescent reaction of these compounds and, hence, favor *T*_1_ chemiexcitation in the increasing order of OH < Cl < Br. If the reported anticancer activity of the compounds is indeed related to the intracellular superoxide-triggered *T*_1_ chemiexcitation, this should lead to heavy-atom-dependent enhancement of their cytotoxicity in the increasing order of OH < Cl < Br. Thus, these three derivatives were subjected to a detailed and in-depth luminometry, photophysical, and theoretical characterization of their chemiluminescent reactions, focused on the possible superoxide-induced generation of triplet excited states. Subsequently, their cytotoxicity was also evaluated for the first time toward neuroblastoma and colon cancer cell lines, with the resulting performance compared with that obtained for breast and prostate cancer. With this approach, we will be able to understand if the heavy-atom effect is indeed responsible for the anticancer activity of these molecules, supporting their identification as prototypical light-free and self-activating photosensitizers, and to understand the scope of their cytotoxicity toward different cancer types.

## 2. Materials and Methods

### 2.1. In Silico Modeling

The *S*_0_ geometry optimization and frequency calculations for OH-Cla dioxetanone were performed with the ωB97XD functional [14] and the 6-31G(d,p) basis set, as well as with an open-shell (U) and broken-symmetry approach. Intrinsic reaction coordinate (IRC) calculations were carried out to assess if the obtained transition state (TS) connects the desired reactants and products. The energies of the *S*_0_ IRC-obtained structures were re-evaluated by single-point calculations with the same functional but using the 6-31 + G (d,p) basis set. The *T*_1_ state was calculated by performing single-point calculations at the same level of theory, on top of the *S*_0_ IRC-obtained structures. ωB97XD is a long-range-corrected hybrid exchange-correlation functional, which provides quite good estimates for π → π * and n → π * local excitation, as well as charge transfer and Rydberg states [15]. Geometry optimizations, frequency calculations, and IRC calculations were made in vacuo, while single-point calculations were made in implicit water using a polarizable continuum model (IEFPCM). All calculations were made using the Gaussian 09 program package [16]. This approach has been used with success before in the study of the chemiexcitation of dioxetanones [9,17].

### 2.2. Synthesis of the Compounds

Br-Cla was prepared following the procedure described in [12], while OH- and Cl-Cla were synthesized through the same synthetic pathway with some modifications (Scheme S1). The detailed synthetic procedure is available in the Supplementary Materials. Briefly, these compounds were obtained through an initial Suzuki-Miyaura cross-coupling between 5-bromopyrazin-2-amine and the corresponding arylboronic acid derivative (Scheme S1). Condensation of the resulting precursor with methyl glyoxal in acid medium yielded the desired reaction product (Scheme S1). High-performance liquid chromatography coupled to a diode array detector (HPLC-DAD), Fourier-transform infrared (FT-IR), and UV-Vis spectroscopy data, as well as details for all compounds, are presented in the Supplementary Materials. ^1^H-NMR and high-resolution mass spectra (HR-MS) for OH-/Cl-Cla are also available in the Supplementary Materials, whereas they were presented in [12] for Br-Cla.

### 2.3. Luminometric and Photophysical Characterization

Chemiluminescence kinetic measurements were performed in a homemade luminometer using a Hamamatsu HC135-01 photomultiplier tube. All reactions took place at room temperature at least in sextuplicate. The light-emitting reactions took place at room temperature at least in sextuplicate and were carried out in either *N*,*N*-dimethylformamide (DMF)-acetate buffer pH 5.14 (0.68%) or methanol in the presence of a superoxide anion source (potassium superoxide, KO_2_). The steady-state chemiluminescent and fluorescent spectra of the three Cla derivatives were measured using a Horiba Jovin Fluoromax 4 spectrofluorimeter, with an integration time of 0.1 s. Slit widths of 5 nm were used for both the excitation and emission monochromators when obtaining fluorescent spectra. Chemiluminescent spectra were obtained with a slit of 29 nm for the emission monochromators. Quartz cells with a 10 mm path length were used.

The light-emitting reactions took place at ambient temperature at least in sextuplicate and were carried out in DMF-acetate buffer pH 5.14 (0.68%) or methanol (in the presence of potassium superoxide (KO_2_)) The chemiluminescent and fluorescent spectra were obtained in 2 mL DMF-acetate buffer pH 5.14 (0.68%) solutions and measured using a Horiba Jovin Fluoromax 4 spectrofluorimeter (The detailed procedure is available in the Supplementary Materials).

### 2.4. Cellular Assays

#### 2.4.1. Cell Lines and Culture

The HT-29 and SH-SY5Y cell lines were obtained from ATCC. The human colon cancer HT-29 cell line was cultured at 37 °C and 5% CO_2_ in McCoy’s 5a Medium Modified supplemented with 10% fetal bovine serum, 100 U/mL penicillin G, and 100 µg/mL streptomycin. Cells were maintained in the logarithmic growth phase and the medium was changed every 3 days. Cells were trypsinized with 0.25% trypsin-EDTA and subcultured in the same medium. SH-SY5Y human neuroblastoma cells were cultured in Dulbecco’s modified Eagle medium (DMEM), supplemented with 100 U/mL penicillin/100 µg/mL streptomycin, and 10% heat-inactivated fetal bovine serum (FBS), incubated at 37 °C in a humidified atmosphere of 95% air and 5% CO_2_. For cell culture maintenance, cells were subcultured once a week and the cell medium was renewed every 2 days. Both the human prostate (PC-3) and the breast (MCF-7) cancer cell lines were cultured in DMEM supplemented with 10% fetal bovine serum (FBS) and 1% antibiotics/antimycotics (complete medium), and they were incubated in a 5% CO_2_ incubator at 37 °C.

#### 2.4.2. Cell Treatment

Before each treatment, HT-29, SH-SY5Y, PC-3, and MCF-7 cells were seeded in 96-well plates in triplicate with the following densities: 5000 cells/well for PC-3 and MCF-7, 15,000 cells/well for HT-29, and 20,000 cells/well for SH-SY5Y. For assays involving HT-29 and SH-SY5Y lines, cells were treated with OH-, Cl-, and Br-Cla (in DMSO) at 0.01, 0.1, 1, 10, 20, 30, 50, 75, or 100 µM for 48 h. During the experimental period, cells were maintained at 37 °C with 5% CO_2_. Controls were composed of DMSO 0.1% *v*/*v*. Cells were exposed to the different treatments for 48h. For assays involving PC-3 and MCF-7 lines, cells were treated with Br-Cla at 0.1, 1, 10, 25, 50, and 75 µM (in methanol) for 72 h [13]. The control was methanol at a maximum final concentration of 0.1% *v*/*v*. The half-maximal inhibitory concentration (IC_50_) value was determined for the different assays by MTT viability assay.

#### 2.4.3. MTT Cytotoxicity Assays

After cell treatment with the Cla derivatives, mitochondrial function was evaluated since mitochondrial dehydrogenases of living cells can reduce the MTT (yellow) to formazans, which are purple compounds. At the end of incubation, the cell medium was removed and 100 µL of MTT solution (0.5 mg/mL in PBS) was added to each well. Cells were then incubated for 3 h, protected from light. After this period, the MTT solution was removed, and DMSO (100 µL/well) was added to solubilize the formazan crystals. Absorbance was measured at 570 nm using an automated microplate reader (Tecan Infinite M200, Tecan Group Ltd., Männedorf, Switzerland). All conditions were performed in triplicate.

#### 2.4.4. Data Analysis and Statistical Analysis

GraphPad Prism 8 (GraphPad Software Inc., San Diego, CA, USA) was used to produce concentration-response curves by nonlinear regression analysis. MTT results are presented as the mean ± SEM for n experiments performed. All data were assessed in three independent experiences. Statistical comparisons between control and treatment groups were performed with one-way ANOVA test. Statistical significance was accepted at a *p*-value < 0.05.

#### 2.4.5. Cell Morphology

Cell morphology was assessed on a Leica DMI 6000B microscope equipped with a Leica DFC350 FX camera and then analyzed with the Leica LAS X imaging software v3.7.4 (Leica Microsystems, Wetzlar, Germany).

## 3. Results and Discussion

### 3.1. In Silico Characterization of R-Cla Derivatives

**Figure 1:**
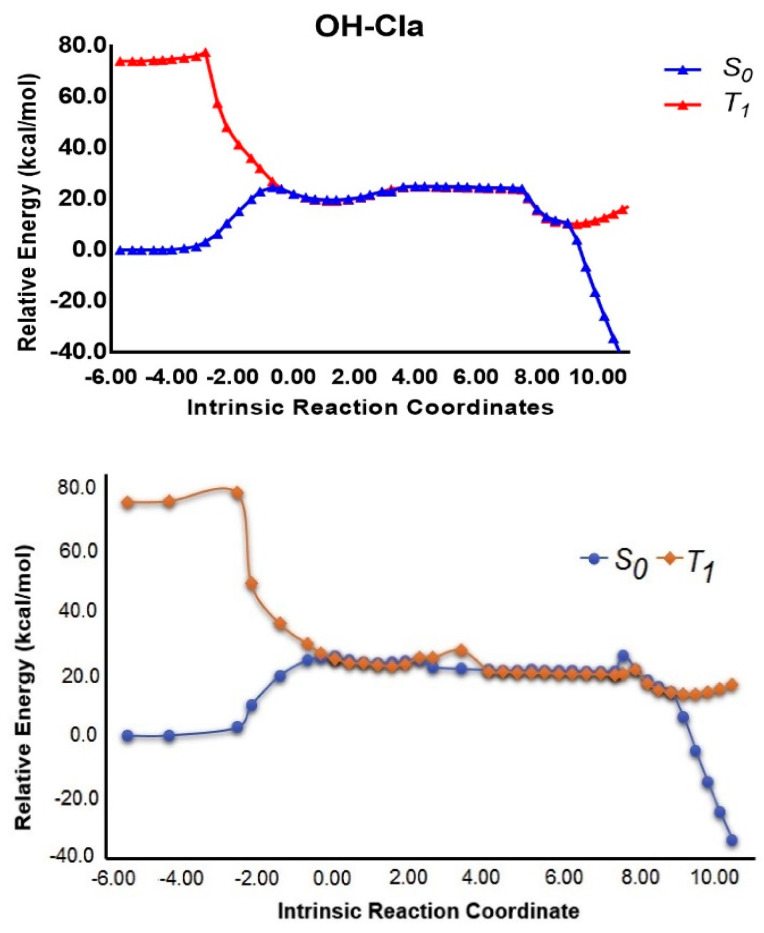
Potential energy curves (in kcal·mol^−1^) for the *S*_0_ and *T*_1_ states during the thermolysis of OH-Cla (top) and Br-Cla dioxetanones (bottom), as a function of intrinsic reaction coordinates (in amu^1/2^ bohr). Calculations were made with the ωB97XD density functional in implicit water. The bottom figure is reprinted with permission from [12]. Copyright Elsevier 2019.

It is important to verify if all the proposed R-Cla derivatives have an intrinsically available pathway for *T*_1_ chemiexcitation during their chemiluminescent reaction, which can be enhanced by the heavy-atom effect, before their synthesis and characterization. Thus, we calculated the potential energy curves for both the *S*_0_ and *T*_1_ states during the thermolysis of OH-Cla (Scheme 1 and Figure 1), with a density functional theory (DFT) approach [11,17]. Similar calculations were previously performed for Br-Cla dioxetanone [12] (Figure 1). Therefore, OH-Cla dioxetanone was chosen to be studied as a reference, since OH-Cla and Br-Cla are on opposite sides regarding the expected enhancement of ISC rate due to the heavy-atom effect.

As consistent with previous reports for different dioxetanones (including Br-Cla) [6,9,11,12,17], the *S*_0_ thermolysis of OH-Cla dioxetanone proceeds via a stepwise biradical mechanism that involves the cleavage of two bonds of the peroxide ring: first by the breaking of the O-O bond, followed by C-C bond cleavage. The *S*_0_ activation energy for the thermolysis reaction of OH-Cla dioxetanone is 24.7 kcal·mol^−1^, only 0.8 kcal·mol^−1^ lower than that of Br-Cla (25.5 kcal mol^−1^) [12]. The interplay between *S*_0_ and *T*_1_ states during the thermolysis reaction (Figure 1) is more important. While, for both dioxetanones, the *S*_0_ – *T*_1_ energy gap is significant at the beginning of the reaction (~75 kcal·mol^−1^), when reaching the TS and onward, both states become degenerated in a large and flat region of the potential energy surface (PES).

This indicates that both chemiluminescent reactions possess an intrinsic pathway for *T*_1_ chemiexcitation, in line with previous studies for this type of system [9,11,12,18]. However, as ISC is a spin-forbidden process, the *S*_0_ → *T*_1_ chemiexcitation pathway for OH-Cla is not expected to be efficient [19]. Given the identical energetic profiles found for OH-Cla and Br-Cla (Figure 1), the addition of halogen atoms of increasing size in Cl-Cla and Br-Cla should only enhance the rate of the ISC process, due to the heavy-atom effect. Thus, this substitution appears to be ideal to assess solely the heavy-atom effect on the possible anticancer activity of these molecules.

It should be noted that an efficient ISC is not the only parameter determining the performance of a photosensitizer in singlet oxygen generation, as it should also have a *T*_1_ state with an energy higher than 0.98 eV (the required energy to convert molecular oxygen into singlet oxygen) [20]. We modeled the adiabatic *S*_0_ – *T*_1_ energy gap of OH-Cla coelenteramide and found a value of 2.78 eV. Furthermore, this value is identical to what was found by us previously for Br-Cla [12]. Thus, both OH-Cla and Br-Cla compounds possess enough energy to generate singlet oxygen. It should be noted that this value was obtained with the M06-2X functional and the 6-31 + G (d,p) basis set, in implicit water, using the SMD model. This approach was used to ensure consistency between studies, as this was the approach used before to calculate the *S*_0_ – *T*_1_ energy gap of Br-Cla coelenteramide [12].

### 3.2. Synthesis of Cla Derivatives

Given that the in silico modeling results indicate that the chemiluminescent reactions of these Cla derivatives present intrinsic pathways for light-free generation of triplet states capable of sensitizing singlet oxygen, we then proceeded to the synthesis of OH-Cla, Cl-Cla, and Br-Cla (Scheme S1). The introduction of heteroatoms with increasing size should enhance the ISC rate due to the heavy-atom effect, thereby allowing us to obtain model molecules with different efficiencies of triplet state generation.

The structures of these compounds were confirmed with ^1^H-NMR spectroscopy (Figures S1–S4) and HR-MS spectrometry (Figures S5–S8) [12]. Complementary analyses by FT-IR spectroscopy showed that the compounds share an intense band in the N-H/C-H stretching region, compatible with highly conjugated heteroaromatics, as well as several bands in the C=C/C=N/C=O bending region. For OH-Cla, minor differences (attributable to the OH moiety) can be observed in the stretching region (Figures S9–S11). HPLC-DAD further confirmed the high purity of these compounds (Figures S12–S14). Analysis by UV-Vis spectroscopy (Figures S15–S17) revealed quite similar absorbance spectra for all compounds, with three bands at ~280 nm, ~350 nm, and ~450 nm. The similarity is expected given that all derivatives present identical molecular structures. Nevertheless, an interesting substitution-related trend was observed, as the relative intensity of the band at ~450 nm (in comparison with the one at ~350 nm) decreased in a relevant manner in the following order: OH > Cl > Br (Figures S15–S17).

### 3.3. Luminometric and Photophysical Characterization

After synthesizing the target derivatives and completing their structural characterization, it is essential to perform their luminometric and photophysical characterization. Specifically, before assessing the potential role of the heavy-atom effect in the anticancer properties of these compounds, it is required to find out if the introduction of heteroatoms of increasing size enhances the *T*_1_ chemiexcitation of these compounds. Thus, we subjected these compounds to a detailed luminometric and photophysical characterization.

**Figure 2:**
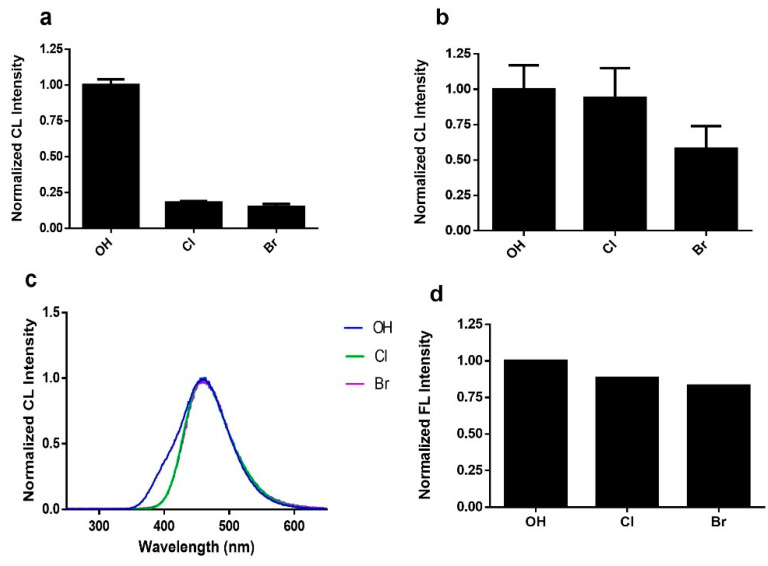
(**a**) Normalized chemiluminescence output of OH-, Cl-, and Br-Cla in DMF-acetate buffer pH 5.14 (0.68%). (**b**) Chemiluminescence intensity of OH-, Cl-, and Br-Cla in the presence of 20 mg of KO_2_ in methanol. (**c**) Normalized chemiluminescence spectra of OH-, Cl-, and Br-Cla in DMF-acetate buffer pH 5.14 (0.68%). (**d**) Normalized fluorescence intensity of spent chemiluminescent reactions of OH-, Cl-, and Br-Cla coelenteramide after 30 min of reaction.

First, we measured the chemiluminescent output of the three Cla derivatives in an aprotic solvent (DMF) with addition of buffer (acetate buffer, pH 5.14), conditions in which Clz and derivates are known to readily generate chemiluminescence by reacting with dissolved oxygen [17]. All compounds emitted chemiluminescence (Figure 2a and Figure S18) with the typical kinetic profile, with a quick rise in light emission on the millisecond timescale and subsequent decay to basal levels (all within 600 ms). Interestingly, there is a clear halogen substitution effect in which the light emitted by Cl- and Br-Cla is significantly lower than that emitted by OH-Cla. Furthermore, among halogenated compounds, the light output decreases in the order of OH > Cl > Br. In solution, triplet states are generally more easily quenched than singlet excited states, thereby not leading to light emission in solution at room temperature. Thus, the quite lower light output of halogenated Cla compounds is indicative of the halogen’s ability to enhance ISC during dioxetanone’s thermolysis, by increasing the triplet-to-singlet product ratio of the studied chemiluminescent reactions [19,21].

One other possible explanation for these variations in intensity could be that the introduction of the halogen atoms decreased the energetic favorability of the *S*_0_ reaction. However, in our previous study of Br-Cla [12], we already found that bromination does not impede the reaction, as it is still highly exothermic (−97.1 kcal·mol^−1^). Furthermore, the *S*_0_ energetics for the thermolysis of OH-Cla and Br-Cla dioxetanones are identical (Figure 1), which means that halogenation should not affect the efficiency of the *S*_0_ chemiluminescent reaction.

**Figure 3:**
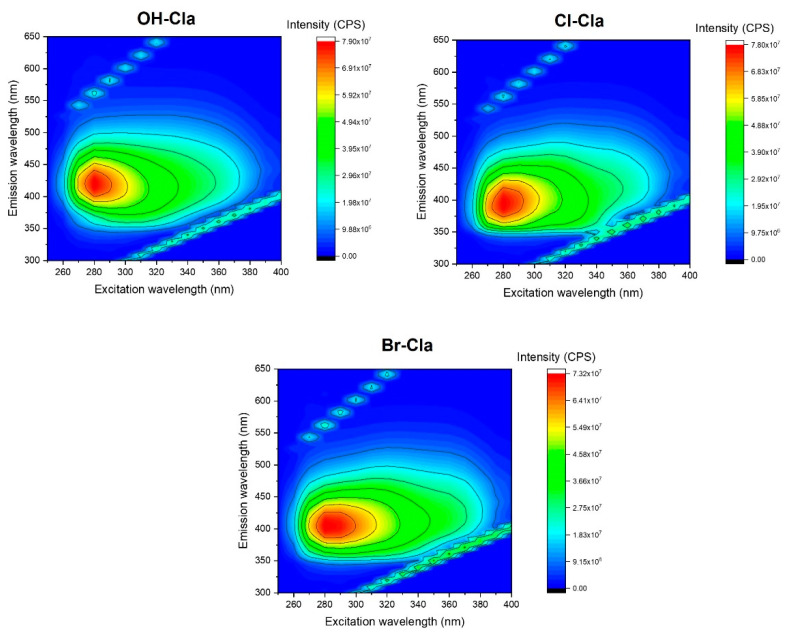
The 2D excitation-emission matrices (EEMs) of spent chemiluminescent reactions of OH-, Cl-, and Br-Cla after 30 min of reaction in DMSO-acetate buffer pH 5.14 (0.68%) solution.

Further support for this conclusion was obtained by analyzing the chemiluminescent kinetic profiles of these compounds (Figure S18). There were no significant differences between compounds, with the kinetic profiles showing identical rises and subsequent decay of light emission in the same timeframe, showing that halogenation does not affect the kinetics of the reaction. The steady-state chemiluminescent spectra (measured during the chemiluminescent reaction in a spectrofluorometer, without the use of an excitation source) were identical for all R-Cla compounds (Figure 2c), with emission maxima at ~480 nm. Lastly, we also measured the 2D excitation-emission matrices (EEMs) for the spent reaction mixtures (30 min after addition to aprotic solvents) of the three compounds (Figure 3). The resulting EEMs were quite similar to each other, with just one emissive center with an excitation wavelength maximum at ~280 nm. The main difference is only a small blue-shift of ~25 nm in the emission maxima between OH-Cla (~425 nm) and Cl-/Br-Cla (~400 nm). Given this high similarity between EEMs for the spent reaction mixtures, we can conclude that halogenation does not affect the outcome of the chemiluminescent reaction in terms of obtained products.

Lastly, a possible explanation for the lower light output, other than the increase in *T*_1_ chemiexcitation, could be that halogenation decreases the singlet emission efficiency of the resulting chemiluminophore (coelenteramide). To verify this hypothesis, we also measured the fluorescence intensity of the spent reaction mixtures (after 30 min of reaction) in an aprotic solvent (Figure 2d and Figure 3). There is indeed a small decrease in emission intensity of the reaction product that correlates with halogen substitution. However, for one, this decrease is significantly lower than that found for the chemiluminescent output (Figure 2a). Furthermore, given that the decrease in emission intensity occurs in the order of OH > Cl > Br, this also indicates a heavy-atom effect. More specifically, this observation is consistent with fluorescence quenching due to ISC enhancement, during the photo-excitation process. Thus, these results also support the conclusion that the decrease in chemiluminescent light output is due to the heavy-atom effect, which enhances the *T*_1_ chemiexcitation and increases the resulting triplet-to-singlet product ratio.

Having demonstrated that the addition of halogens enhances the production of triplet states, it is also important to assess the ability of the superoxide anion to trigger the chemiluminescent reaction of the studied molecules. This type of ROS is overexpressed in cancer cells [22,23]; thus, it could be thought of as a cancer marker inducing chemiluminescence and a tumor-selectivity profile to our molecules. It should be noted that we previously demonstrated our compounds as having selectivity for breast cancer, as Br-Cla induced significant toxicity toward breast cancer cell lines without affecting non-cancer cell lines in the same concentration range [12].

The ability of superoxide anions to trigger the chemiluminescent reaction of these compounds was demonstrated by measuring their chemiluminescent output, after KO_2_ addition (a known superoxide anion source) in methanol. Interestingly, all compounds responded to the addition of KO_2_ with immediate emission of light and subsequent decay to basal levels (Figure S19). The kinetics of this reaction is significantly quicker than in aprotic solvents, but this difference can be attributed to the instability of KO_2_ in solution. The more relevant result is that all compounds responded similarly to superoxide anion, meaning that they can be activated by a cancer marker. It should also be pointed out that the measured chemiluminescent output showed a substitution-induced effect, in the order OH > Cl > Br (Figure 2b). Once again, this points to halogen substitution increasing the triplet-to-singlet ratio.

### 3.4. In Vitro Cytotoxic Activity of R-Cla

The next step of this study was the evaluation of the in vitro anticancer activity of these compounds. Both the in silico modeling and the luminometric/photophysical characterization showed that the introduction of halogens enhances the superoxide anion-triggered generation of triplet states (with enough energy to sensitize singlet oxygen), without affecting the chemiluminescent reaction. Thus, if the anticancer activity found before for this type of molecules [12,13] is indeed related to a light-free and self-activating photodynamic effect, we should see a halogen-dependent effect on their anticancer activity.

Given this, the potential anticancer activity of the three R-Cla compounds was evaluated in both colon cancer (HT-29) and neuroblastoma (SH-SY5Y) cell lines with a standard MTT assay for exposure times of 48h, along with changes in cell morphology analyzed by microscopy.

**Figure 4:**
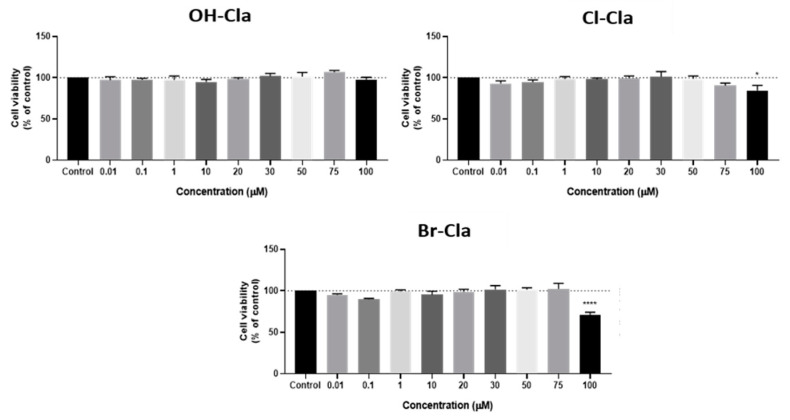
Effect of OH-, Cl-, and Br-Cla on HT-29 cell viability. Cells were cultured in the presence of increasing concentrations of each compound. After 48 h, an MTT assay was performed to measured cellular viability. Results are presented as mean ± SEM. * Statistically significant vs. control at *p* < 0.05; **** statistically significant vs. control at *p* < 0.0001.

**Figure 5:**
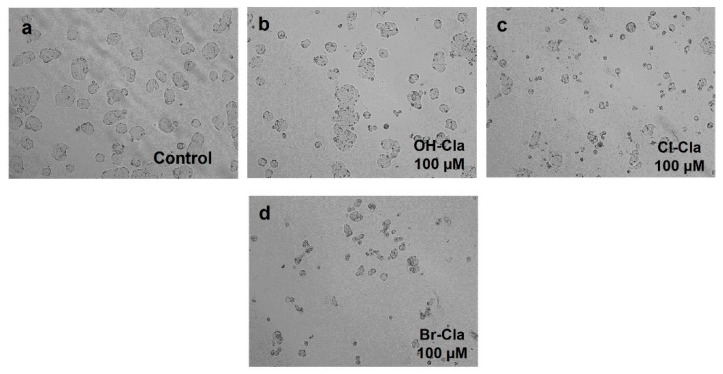
Microscopic cellular visualization of HT-29 cells after 48 h of incubation with cell medium and (**a**) 0.1% DMSO (control), (**b**) OH-Cla, (**c**), Cl-Cla, or (**d**) Br-Cla. Representative images were obtained with a high contrast brightfield objective (10×) (LionHeart FX Automated Microscope), from three independent experiments.

OH-Cla did not have any effect on the viability of colon cancer cells (Figure 4) and did not cause any changes in their morphology (Figure 5b). This indicates that OH-Cla has no anticancer activity toward these cells, which is expected since, without the heavy-atom effect introduced by the halogens, there is no reason for efficient *T*_1_ chemiexcitation. Interestingly, while Cl-Cla induced virtually no anticancer effect in these cells, there was a statistically significant difference at a concentration of 100 µM (Figure 4). In addition, microscopic analysis at the two highest concentrations indicated that the cells were rounder and in lesser number, without the formation of aggregates (Figure 5c). As for Br-Cla, there was a relevant decrease in cellular viability at the highest concentration (100 µM) (Figure 4), with the morphological analysis showing a clear decrease in the number of cells at the highest concentration, as well as a change in the size and shape of the cells, which were smaller and rounder (Figure 5d).

These results are interesting because the observed anticancer activity toward HT-29 cell lines was low, while we could observe a distinct halogen-dependent effect. Specifically, OH-Cla did not present any activity, while Cl-Cla and Br-Cla presented increasing activity. This is a first demonstration of the heavy-atom effect, which indicates that this anticancer activity is indeed related to a light-free and self-activating photodynamic effect.

It should also be noted that a positive control test was performed by exposing these cells to an antineoplastic drug commonly used in colon cancer therapy, 5-fluorouracil (5-FU), in the same concentration range as R-Cla (Figure S20). This drug induced a significant decrease in cell viability for all concentrations, while microscopic evaluation revealed that all cells appeared to be morphologically changed (Figure S21a–c).

**Figure 6:**
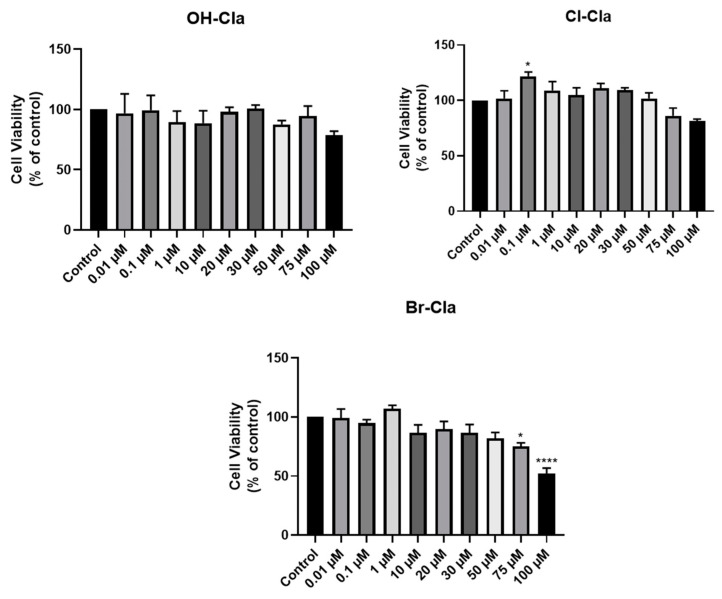
Effects of OH-, Cl-, and Br-Cla on SH-SY5Y cellular viability. Cells were cultivated in the presence of increasing concentrations of each compound. After 48 h, an MTT assay was performed to measured cellular viability. Results are presented as mean ± SEM. * Statistically significant vs. control at *p* < 0.05; **** statistically significant vs. control at *p* < 0.0001.

**Figure 7:**
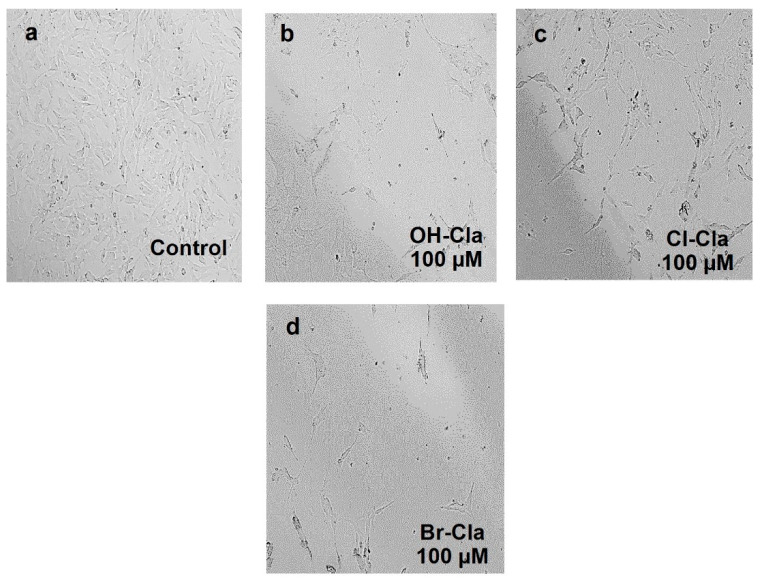
Microscopic cellular visualization of SH-SY5Y cells after 48 h of incubation with cell medium and (**a**) 0.1% DMSO (control), (**b**) OH-Cla, (**c**), Cl-Cla, or (**d**) Br-Cla. Representative images were obtained with a high contrast brightfield objective (10×) (LionHeart FX Automated Microscope), from three independent experiments.

In neuroblastoma cells (Figure 6 and Figure 7), the results were similar and neither OH-Cla nor Cl-Cla altered cell viability in a relevant manner. On the contrary, Br-Cla (Figure 6 and Figure 7d) induced a significant decrease in cell viability, although not as marked as for 5-FU (Figures S20b and S22), which was also used as a positive control for neuroblastoma cells. This compound induced a sharp decrease in cell viability. The cell viability results were consistent with microscopic evaluation (Figure 7 and Figure S22).

Given this, it is clear that there was a halogen-dependent effect on the anticancer activity of these compounds for both cancer cell lines. This is in line with our proposition that the addition of halogens to Clzs will provide them with anticancer activity, by enhancing *T*_1_ chemiexcitation and the subsequent intracellular generation of triplet states capable of sensitizing singlet oxygen [12,13]. In conclusion, our results indicate that the anticancer activity of our Clz derivatives [12,13] is indeed related to the heavy-atom-induced triplet state generation during their chemiluminescent reaction. Therefore, it appears that we can consider our compounds as prototypical single-molecule-photosensitizers capable of an intracellular and self-activating photodynamic effect, which is triggered by a cancer marker.

Having said that, it is also important to note that the intrinsic anticancer activity of the studied compounds (including Br-Cla) might not be as high as desired toward neuroblastoma and colon cancer cell lines, especially when compared to 5-FU. Given this, it is important to determine whether the magnitude of the obtained anticancer activity is similar across different cancer types or if it is dependent on the studied cell line.

To clarify this topic, we calculated for the first time the IC_50_ of Br-Cla for different cell lines. Br-Cla was chosen for its consistent toxicity in this study, contrary to OH-Cla and Cl-Cla. IC_50_ values were determined for neuroblastoma SH-SY5Y, prostate PC-3, and breast MCF-7 cancer cell lines (Table 1). We did not include assays with the HT-29 cancer cell line, due to the limited toxicity of Br-Cla toward it (Figure 4). We included assays with PC-3 and MCF-7 cell lines due to previous promising results of Br-Cla and other Clz derivatives toward them [12,13]. For the assays with these two cell lines, we maintained the conditions employed before for consistency purposes [13].

**Figure 8:**
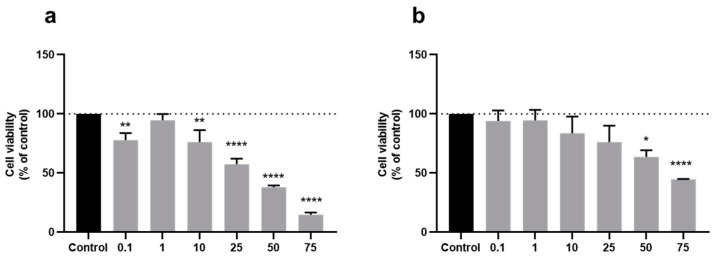
Relative viabilities of (**a**) MCF-7 and (**b**) PC-3 cells after 72 h incubation with several concentrations of Br-Cla, always without light irradiation. Results are presented as mean ± SEM. * Statistically significant vs. control at *p* < 0.05; ** statistically significant vs. control at *p* < 0.01; **** statistically significant vs. control at *p* < 0.0001.

The impact of treatment with Br-Cla on the cellular viability of PC-3 and MCF-7 cell lines can be found in Figure 8. The anticancer activity of Br-Cla was improved in these two cell lines; in breast MCF-7 cancer cell lines, there was a noticeable decrease in cell viability from 10 μM onward, whereas, in prostate PC-3 cancer cells, Br-Cla decreased the cell viability from 25 μM onward, achieving toxicity values higher than 50% at a concentration of 75 μM. This improved efficiency was confirmed by the determined IC_50_ values (Table 1). More specifically, for SH-SY5Y, the obtained IC_50_ was more than double the values found for the other cell lines. Interestingly, the IC_50_ values found for PC-3 and MCF-7 cells were similar, although slightly lower for the latter cell line. Furthermore, to discard the hypothesis that these differences could be related to the different duration of treatment with Br-Cla, we also determined the IC_50_ for the MCF-7 cell line with 24 h treatment (Table 1). This value (33.84 μM) was found to be significantly higher than that found for 72 h treatment (21.56 μM), which indicates that increasing the incubation time increased the obtained IC_50_. More important is that the IC_50_ found for SH-SY5Y (50.92 μM) was still significantly higher than the IC_50_ found for MCF-7, irrespective of the duration of treatment with Br-Cla.

Thus, the results indicate that it is safe to state that the magnitude of the anticancer activity of Br-Cla is dependent on the cancer cell type, being higher for prostate and breast cancer than for neuroblastoma and colon cancer. Further studies are required to assess if these differences arise from (among other possibilities) higher resistance of HT-29 and SH-SY5Y cell lines to the photodynamic effect, lower efficiency of internalization of R-Cla compounds into these cell lines, or a lower generation of superoxide anion in these cells.

## 4. Conclusions

In conclusion, we reported the target-oriented synthesis of three Clz derivatives (OH-Cla and its halogenated Cl- and Br- derivatives), to provide anticancer activity to this class of compounds through the heavy-atom effect. On the basis of this strategy, we developed novel compounds able to directly generate readily available triplet excited states with enough energy to sensitize singlet oxygen, in a chemiluminescent reaction triggered by a cancer marker. This was achieved by the introduction of the heavy-atom effect into this system, which enhanced the ISC rate of available *S*_0_ → *T*_1_ chemiexcitation pathways. The anticancer activity of these compounds was evaluated toward different cancer cell lines, and a clear halogen effect was observed with Cl-Cla and Br-Cla presenting increasing toxicities. Furthermore, it was found that the magnitude of the anticancer activity of these types of compounds is dependent on the cancer cell line, being more relevant in prostate and breast cancer than in neuroblastoma and colon cancer. Thus, the results indicate that applying the heavy-atom effect to marine Clz would be a promising strategy for designing self-activating and light-free photosensitizers. With this design strategy, the Clz system appears to be a prototypical system for optimized photosensitizers to be used in PDT strategies not limited by the need for external light sources.

## 5. Patents

Patent PCT/IB2019/053642 (pending)—chemiluminescent imidazopyrazinone-based photosensitizers with available singlet and triplet excited states.

## Figures and Tables

**Table 1:** IC_50_ (in μM) values for Br-Cla in neuroblastoma SH-SY5Y, prostate PC-3, and breast MCF-7 cancer cell lines. Assays were performed with either 48 (SH-SY5Y) or 72 h (PC-3 and MCF-7) [13].

Molecule	SH-SY5Y	PC-3	MCF-7
Br-Cla	50.92	24.28	21.56 (33.84) ^1^

^1^ The value within parentheses refers to 24 h treatment.

## Supplementary Materials

The following are available online at https://www.mdpi.com/article/10.3390/biomedicines9091199/s1: Scheme S1. General synthetic procedure for the synthesis of the coelenterazine analogs; Figure S1. 1H-NMR spectrum of 2a in CDCl3; Figure S2. 1H-NMR spectrum of 2b in CDCl3; Figure S3. 1H-NMR spectrum of OH-Cla in MeOH d4; Figure S4. 1H-NMR spectrum of Cl-Cla in MeOH d4; Figure S5. ESI-MS (+) spectrum of 2a; Figure S6. ESI-MS (+) spectrum of 2b; Figure S7. HR-MS ESI (−) spectrum of OH-Cla; Figure S8. HR-MS ESI (+) spectrum of Cl-Cla; Figure S9. FT-IR spectrum of OH-Cla; Figure S10. FT-IR spectrum of Cl-Cla; Figure S11. FT-IR spectrum of Br-Cla; Figure S12. HPLC chromatogram and DAD-UV/Vis spectrum of OH-Cla; Figure S13. HPLC chromatogram and DAD-UV/Vis spectrum of Cl-Cla; Figure S14. HPLC chromatogram and DAD-UV/Vis spectrum of Br-Cla; Figure S15. Absorbance spectra of OH-Cla in MeOH:CH3CN (1:1) and CH3CN:water:formic acid (3:2:0.1); Figure S16. Absorbance spectra of Cl-Cla in MeOH:CH3CN (1:1) and CH3CN:water:formic acid (3:2:0.1); Figure S17. Absorbance spectra of Br-Cla in MeOH:CH3CN (1:1) and CH3CN:water:formic acid (3:2:0.1); Figure S18. Normalized chemiluminescence profiles of OH-, Cl-, and Br-Cla in DMF-acetate buffer pH 5.14 (0.68%); Figure S19. Normalized chemiluminescence profiles of OH-, Cl-, and Br-Cla in the presence of 15 mg of KO_2_ in methanol; Figure S20. Cellular viability assays of HT-29 (a) and SH-SY5Y (b) cells after 48 h of incubation with 5-FU; Figure S21. Microscopic cellular visualization of HT-29 cells after 48 h of incubation with 5-FU at (a) 0.01 µM, (b) 20 µM, and (c) 100 µM; Figure S22. Microscopic cellular visualization of SH-SY5Y cells after 48 h of incubation with 5-FU at (a) 10 µM and (b) 100 µM.
