# Supplementary material for: Target-Oriented Synthesis of Marine Coelenterazine Derivatives with Anticancer Activity by Applying the Heavy-Atom Effect
Source: Biomedicines. 2021 Sep 11;9(9):1199. doi: 10.3390/biomedicines9091199 (PMC8467094; doi:10.3390/biomedicines9091199)
Supplement: Supplementary file 1 [file biomedicines-09-01199-s001.zip › biomedicines-1335325-supplementary.pdf]

Supplementary Information for

Target-Oriented Synthesis of Marine Coelenterazine Derivatives with Anticancer Activity by Applying the Heavy-Atom Effect

Carla M. Magalhães [1], Patricia González-Berdullas [1], Diana Duarte [2,3], Ana Salomé Correia [2,3], José E. Rodríguez-Borges [4], Nuno Vale [2,3], Joaquim C. G. Esteves da Silva [1,2] and Luís Pinto da Silva [1,5,*]

1 Chemistry Research Unit (CIQUP), Faculty of Sciences, University of Porto, R. Campo Alegre 687, 4169-007 Porto, Portugal; up201201533@edu.fc.up.pt (C.M.M.); patricia.berdullas@fc.up.pt (P.G.-B.); jcsilva@fc.up.pt (J.C.G.E.d.S.)

2 OncoPharma Research Group, Center for Health Technology and Services Research (CINTESIS), Rua Dr. Plácido da Costa, 4200-450 Porto, Portugal; dianaduarte29@gmail.com (D.D.); anncorr07@gmail.com (A.S.C.); nunovale@med.up.pt (N.V.)

3 Department of Community Medicine, Health Information and Decision (MEDCIDS), Faculty of Medicine, University of Porto, Alameda Prof. Hernâni Monteiro, 4200-319 Porto, Portugal

4 LAQV/REQUIMTE, Department of Chemistry and Biochemistry, Faculty of Sciences, University of Porto, R. Campo Alegre 697, 4169-007 Porto, Portugal; jrborges@fc.up.pt

5 LACOMEPHI, GreenUPorto, Department of Geosciences, Environment and Territorial Planning, Faculty of Sciences, University of Porto, R. Campo Alegre 697, 4169-007 Porto, Portugal

* Correspondence: luis.silva@fc.up.pt

Table of Content

1. Experimental Section **S1**

1.1. General methods S1

1.2. Instruments and data analysis S1

1.2.1. NMR *S1*

1.2.2. HR-MS *S2*

1.2.3. ESI-MS *S2*

1.2.4. FT-IR Spectra *S2*

1.2.5. UV-Vis Spectra *S2*

1.2.6. HPLC-DAD *S2*

1.3. Synthesis and characterization of **OH-**, **Cl-**, and **Br-Cla** S3

*1.3.1. General procedure for the synthesis of (**2**)* *S3*

*1.3.2. General procedure for the synthesis of **R-Cla*** *S4*

1.4. Photophysical Characterization S4

2. Supporting figures **S6**

2.1. NMR Spectra S6

2.2. Mass Spectra S8

2.3. FT-IR Spectra S10

2.4. HPLC-DAD S11

2.5. Absorbance spectra S13

2.6. Photophysical characterization S14

2.7. Cellular Assays S15

1. Experimental Section

1.1. General methods

Reagents and solvents were purchased from Merck and used without further purification. All reactions involving oxygen or moisture-sensitive compounds were carried out under dry nitrogen atmosphere. Ice-water and silicon baths were used for reactions at low and high temperatures, respectively, with all reaction temperatures referring to the external bath. Organic extracts were dried over anhydrous Na_2SO_4, filtered and concentrated using a rotary evaporator (Büchi® Rotavapor® R-210, Büchi® B-491 Heating Bath 120V, KNF Neuberger D-79112 Vacuum Pump N 035.1.2 AN.18).

Reactions were monitored by thin-layer chromatography (TLC) using aluminum-backed Merck 60 F_{254} silica gel plates and ethyl/acetate n-hexanes solvent systems. After visualization under ultraviolet light at 254 nm and 365 nm, the plates were developed by immersion in a solution containing a mixture of p-anisaldehyde (2.5%), acetic acid (1%), and sulfuric acid (3.4%) in 95% ethanol followed by heating. Purification of the compounds was carried out by trituration (hexanes or Et_2O) or column chromatography using silica gel (Aldrich, 230-400 mesh) and EtOAc-hexanes mixtures. Compounds were systematically named and numbered following IUPAC recommendations with ChemDraw 20.0.0.41.

McCoy's 5A Medium powder (modified with *L*-glutamine, without sodium bicarbonate), fetal bovine serum (FBS) and penicillin-streptomycin solution were purchased from Millipore Sigma (Merck KGaA, Darmstadt, Germany). Other cell culture reagents were purchased from Gibco (Thermo Fisher Scientific, Inc, Waltham, MA, USA). Thiazolyl Blue Tetrazolium Bromide (MTT, cat. no. M5655) and 5-fluorouracil (**5-FU**, cat. no. F6627) were obtained from Sigma-Aldrich (Merck KGaA, Darmstadt, Germany).

1.2.Instruments and data analysis

1.2.1. *NMR*

NMR spectra were recorded in $CDCl_3$ or CD_3OD solutions on a Bruker NMR spectrometer (Bruker Advance III 400 MHz Ascend, 9.4 Tesla), and chemical shifts are reported on the δ scale (ppm) using the residual solvent signals [δ = 7.26 ppm (^1H, $CDCl_3$), δ = 77.0 ppm (^{13}C, t, $CDCl_3$); δ = 4.870 ppm (^1H, Methanol-d_4), δ = 49.0 ppm (^{13}C, hept, Methanol-d_4)] as internal standards. Coupling constants (*J*) are reported in Hz.

1.2.2. *HR-MS*

HR-MS analysis was done on a LTQ OrbitrapTM XL hybrid mass spectrometer (Thermo Fischer Scientific, Bremen, Germany) controlled by LTQ Tune Plus and Xcalibur 2.1.0.

1.2.3. *ESI-MS*

Direct injection ESI-MS was made using a Thermo Finnigan™ LCQ™ Deca XP Max (Thermo Electron Corporation, Waltham, USA) mass spectrometer. This device consits on electrospay interface as ionization source and a quadruple ion trap for MS^n experiments. It was operated as follows: spray voltage, 5 kV; capillary voltage, ±15 V; capillary temperature, 300 ºC.

1.2.4. *FT-IR Spectra*

FT-IR spectra were measured in a JASCO FT/IR-4600 (JASCO Corp., Tokyo, Japan) equipped with ATR-Pro One accessory, between 449.3-4000.6 cm^{-1} (data interval, 0.964233 cm^{-1}; resolution, 4 cm^{-1}; accumulation, 16 scans; aperture, 7.1 mm; scanning speed, 2 mm sec^{-1}; filter, 30000 Hz).

1.2.5. *UV-Vis Spectra*

The absorbance spectra were measured using a VWR® Spectrophotometer (UV-3100PC), and a quartz cuvette Micro Cell 115F-QS LP with a 10x2mm light path (Hellma Analytics). Solvents were purchased from Merck and were HPLC-grade.

1.2.6. *HPLC-DAD*

The reverse-phase (RP) HPLC-DAD chromatographic system consisted of a Thermo Scientific SpectraSystem P1000 Isocratic Pump coupled to a Thermo Finnigan UV6000 LP diode array detector, with a Rheodyne manual injection valve and a Hypersil GOLD (5 µm, 150 x 2.1 mm) column using 1:1 MeOH:ACN (Flow rate: 0.80 mL/min) as the mobile phase.

1.3. Synthesis and characterization of OH-, Cl-, and Br-Cla

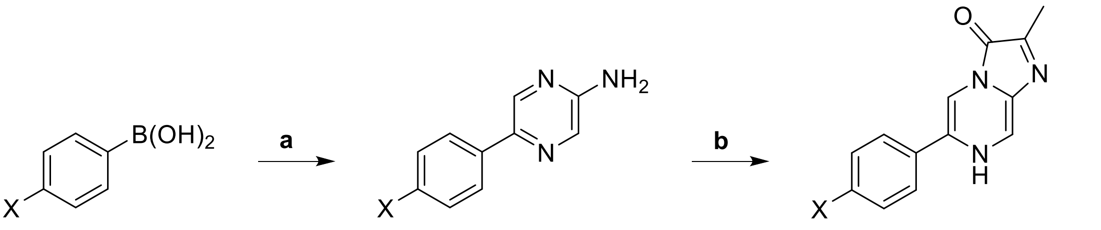

1a: X= OTBS
1b: X= Cl
1c: X= Br

2a: X= OTBS (92%)
2b: X= Cl (87%)
2c: X= Br (85%)

OH-Cla: X= OH (88%)
Cl-Cla: X= Cl (86%)
Br-Cla: X= Br (84%)

a. 5-bromopyrazin-2-amine, K_2CO_3 (1M), $PdCl_2(PPh_3)_2$, THF/H_2O, reflux. **b.** 2-Oxopropanal, $HCl_{(aq)}$, EtOH, reflux.

Scheme S1. General synthetic procedure for the synthesis of the coelenterazine analogs.

1.3.1. *General procedure for the synthesis of (2)*

An aqueous solution of K_2CO_3 (1 M, 11.25 mmol, 7.5 equiv) was added to a solution of the corresponding phenylboronic acid derivative (**1**) (1.5 mmol, 1 equiv) and 5-bromopyrazin-2-amine (0.261 g, 1.5 mmol, 1 equiv) in THF (15 mL) and was deoxygenated with N_2. Then $PdCl_2(PPh_3)_2$ (0.053 g, 0.075 mmol, 0.05 equiv) was added and the resulting mixture was stirred at 80 ºC (external bath temperature) until no starting material was detected by TLC (1:1 EtOAc-hexanes). The reaction mixture was cooled to room temperature and the aqueous phase discarded. The combined organic layers were washed with brine, dried, concentrated under reduced pressure, and filtered through a pad of celite, eluting with EtOAc. The resulting solution was concentrated under reduced pressure to give (**2**), which was triturated with hexanes and vacuum-dried.

- **5-[4-((tert-Butyldimethylsilyl)oxy)phenyl]pyrazin-2-amine (2a)**

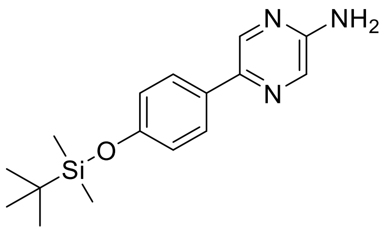

92%, R_f = 0.42 (50% EtOAc/hexanes), yellow solid. **^{1}H NMR** (400 MHz, $CDCl_3$) δ 8.35 (s, 1H), 7.99 (s, 1H), 7.73 (d, *J* = 8.6 Hz, 2H), 6.90 (d, *J* = 8.6 Hz, 2H), 4.83 (bs, 2H), 0.98 (s, 9H), 0.20 (s, 6H). **ESI-MS (+)**: m/z: calcd for $[C_{16}H_{24}N_3OSi]^+$: 302.1689 $[M+H]^+$; found 302.5333 $[M+H]^+$.

- **5-(4-Chlorophenyl)pyrazin-2-amine (2b)**

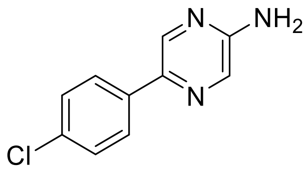

87%, R_f = 0.24 (50% EtOAc/hexanes), yellow solid. **^{1}H NMR** (400 MHz, $CDCl_3$) δ 8.42 (d, *J* = 1.5 Hz, 1H), 8.06 (d, *J* = 1.6 Hz, 1H), 7.92 – 7.73 (m, 2H), 7.46 – 7.37 (m, 2H), 4.66 (bs, 2H). **ESI-MS (+)**: m/z: calcd for $[C_{10}H_9ClN_3]^+$:

206.0485 [M+H]$^+$; found 206.5333 [$C_{10}H_9{}^{35}ClN_3$]$^+$, 208.5333 [$C_{10}H_9{}^{37}ClN_3$]$^+$.

1.3.2. *General procedure for the synthesis of R-Cla*

A solution of **(2)** (1 mmol, 1 equiv) and methylglyoxal (1.5 mmol, 1.5 equiv) in EtOH (9 mL) was deoxygenated with N_2. Then the resulting mixture was cooled to 0 ºC, HCl (37%, 3.6 mmol, 3.6 equiv) was added, the solution was stirred up to room temperature, and then stirred at 80 ºC until no starting material was detected by TLC (1:1 EtOAc-hexanes). The reaction mixture was cooled to room temperature and the resulting solution was concentrated under reduced pressure to give **(R-Cla)**, which was redissolved in the minimum amount of EtOAc, precipitated with diethyl ether, and vacuum-dried.

- **6-(4-Hydroxyphenyl)-2-methylimidazo[1,2-*a*]pyrazin-3(7*H*)-one (OH-Cla)**

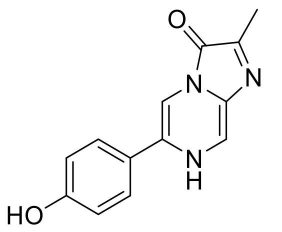

88%, burgundy solid. **^{1}H NMR** (400 MHz, MeOD) δ 9.04 (d, *J* = 1.3 Hz, 1H), 8.54 (d, *J* = 1.3 Hz, 1H), 7.88 – 7.82 (m, 2H), 7.00 – 6.95 (m, 2H), 2.58 (s, 3H). **HRMS** (ESI-TOF$^-$): m/z: calcd for [$C_{13}H_{10}N_3O_2$]$^-$: 240.0779 [M-H]$^-$; found 240.0750.

- **6-(4-Chlorophenyl)-2-methylimidazo[1,2-*a*]pyrazin-3(7*H*)-one (Cl-Cla)**

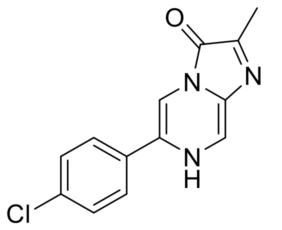

86%, brown solid. **^{1}H NMR** (400 MHz, MeOD) δ 9.11 (s, 1H), 8.76 (s, 1H), 8.10 – 8.05 (m, 2H), 7.62 – 7.57 (m, 2H), 2.58 (s, 3H). **HRMS** (ESI-TOF$^+$): m/z: calcd for [$C_{13}H_{11}N_3O_2$]$^+$: 260.0591 [M+H]$^+$; found 260.0593.

1.4.Photophysical Characterization

The light-emitting reactions took place at ambient temperature at least in sextuplicate and were carried out in DMF-acetate buffer pH 5.14 (0.68%). The reaction was initiated by the injection of DMF-acetate buffer pH 5.14 (0.68%) into an assay tube containing the **OH-**, **Cl-**, or **Br-Cla**, resulting in a final volume of 500 µL and a concentration of chemiluminescent substrate of 10 µM. The light-emitting reactions in the presence of potassium superoxide(KO_2) were carried out in methanol. The reaction was initiated by the injection of methanol and **OH-**, **Cl-**, or **Br-Cla** (10µM) into an assay tube containing 15, 20, or 25 mg of KO_2, resulting in a final volume of 500 µL.

The chemiluminescent spectra were obtained in 2 mL DMF-acetate buffer pH 5.14 (0.68%) solutions, and were measured immediately after the addition of aprotic solvent. The spectra were measured in the above-mentioned spectrofluorimeter with a slit of 29 nm for the emission monochromators, and with the excitation source turned off. The fluorescent spectra of the spent chemiluminescent reaction mixtures were measured after 30 minutes of reaction, after which the chemiluminescent reaction was complete.

The absorbance spectra were measured between 250-600 nm, with 1 nm steps. Stock solutions of the compounds were prepared by dissolving 2 mg of the compounds in 5 mL of MeOH, and then stored at -20º C until use. 30µM solutions were prepared for each compound from stock solution. Solvents were purchased from Merck and were HPLC-grade. Purity of the compounds was estimated by HPLC-DAD. 20µM methanolic solutions were prepared for each compound from stock solution.

2. Supporting figures

2.1. NMR Spectra

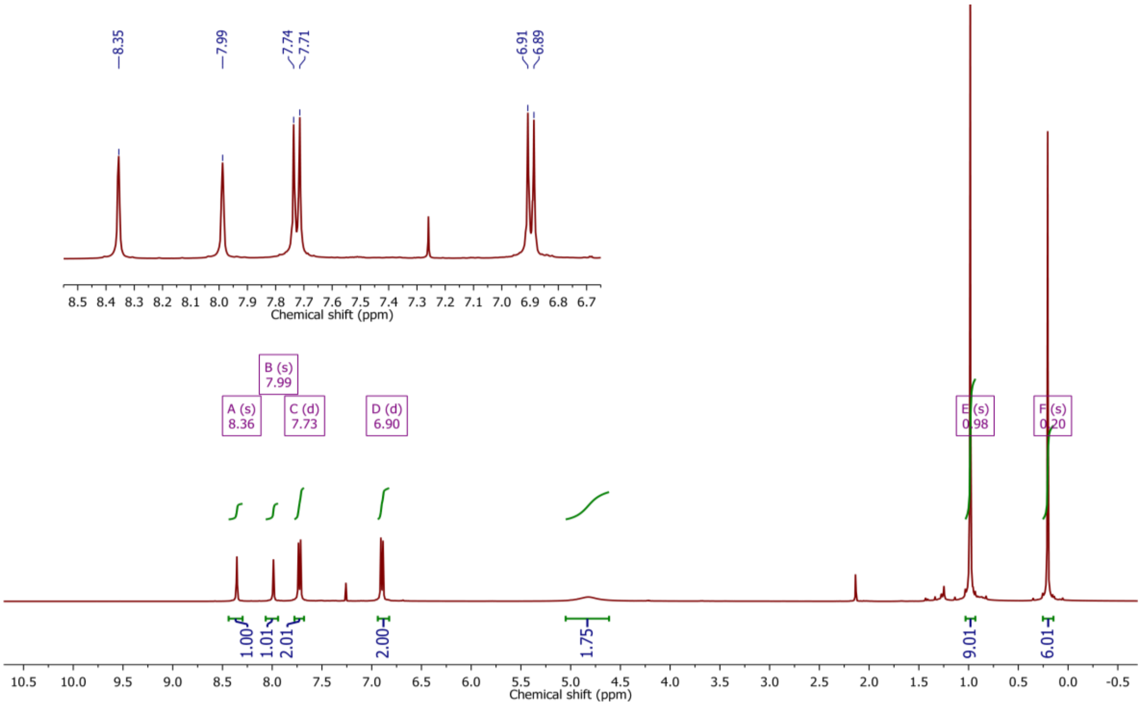

Figure S1. ^{1}H-NMR spectrum of 5-[4-((*tert*-butyldimethylsilyl)oxy)phenyl]pyrazin-2-amine (**2a**) in $CDCl_3$.

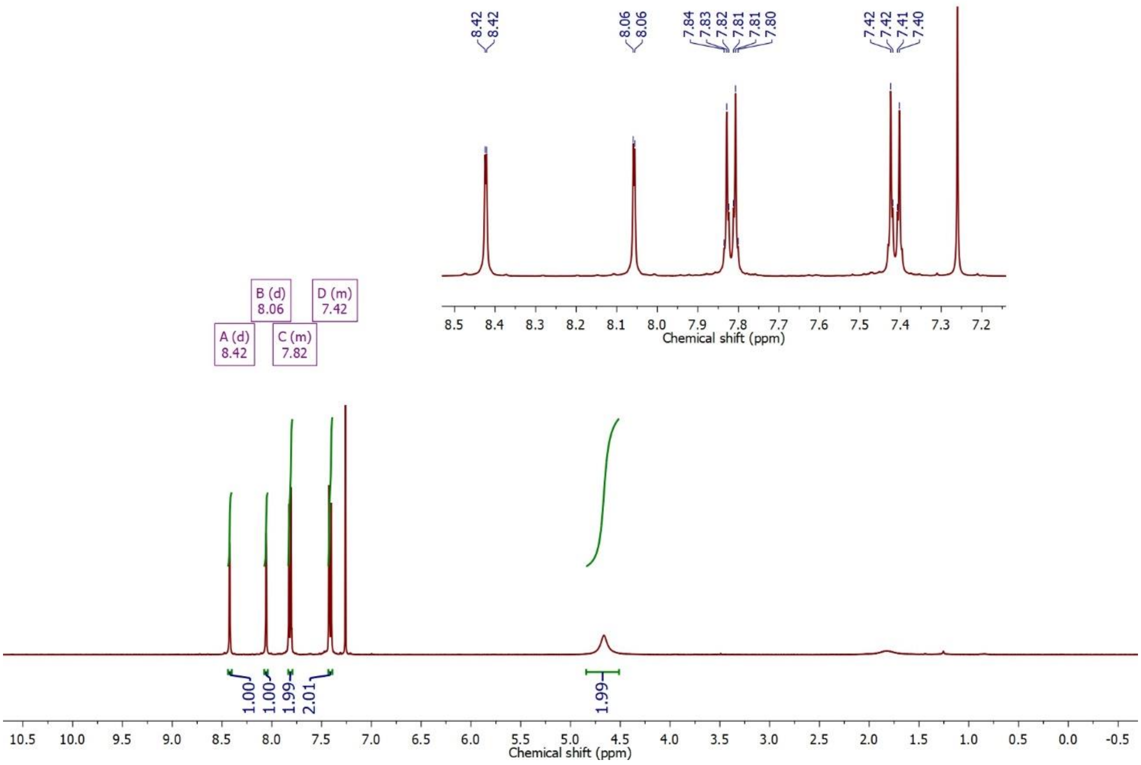

Figure S2. ^{1}H-NMR spectrum of 5-(4-chlorophenyl)pyrazin-2-amine (**2b**) in $CDCl_3$.

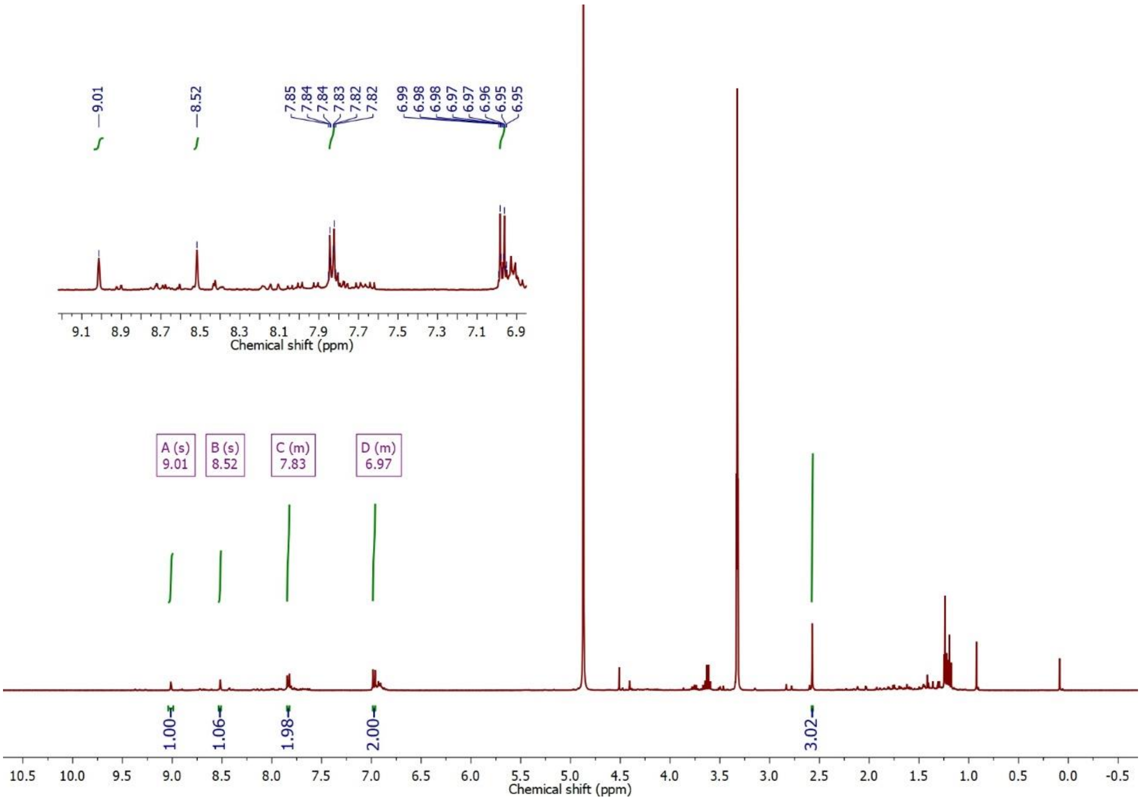

Figure S3. ^{1}H-NMR spectrum of 6-(4-hydroxyphenyl)-2-methylimidazo[1,2-*a*]pyrazin-3(7*H*)-one (**OH-Cla**) in MeOH d_4.

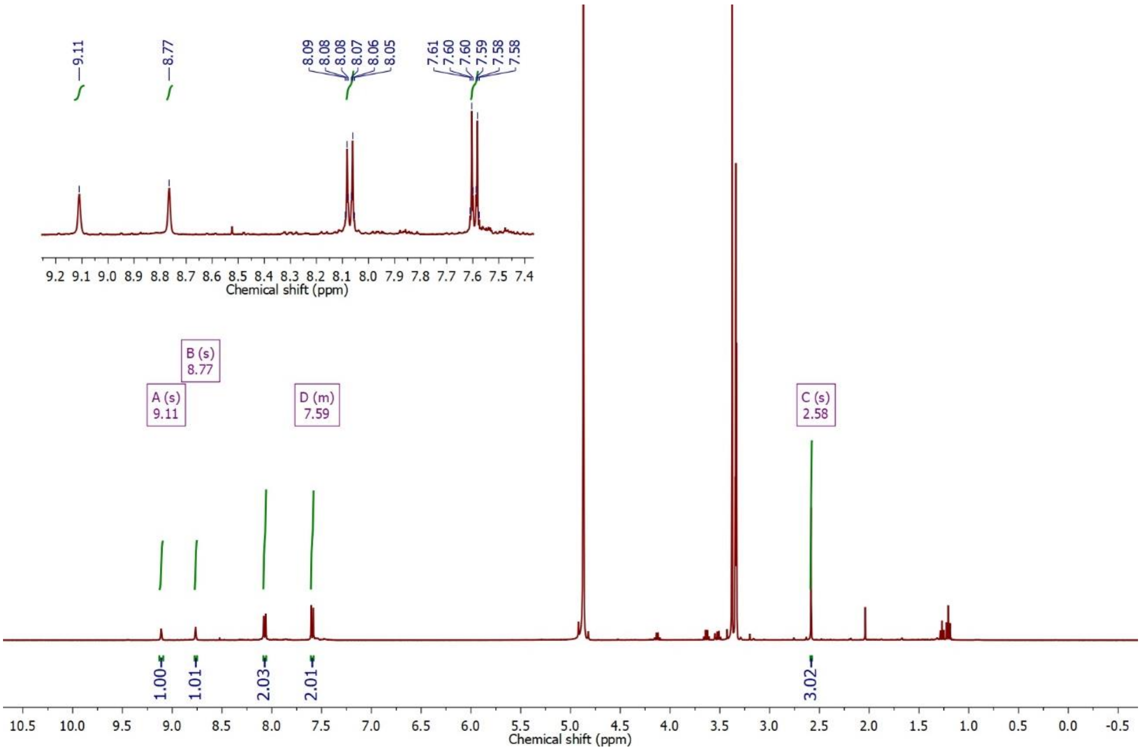

Figure S4. ^{1}H-NMR spectrum of 6-(4-chlorophenyl)-2-methylimidazo[1,2-*a*]pyrazin-3(7*H*)-one (**Cl-Cla**) in MeOH d_4.

2.2.Mass Spectra

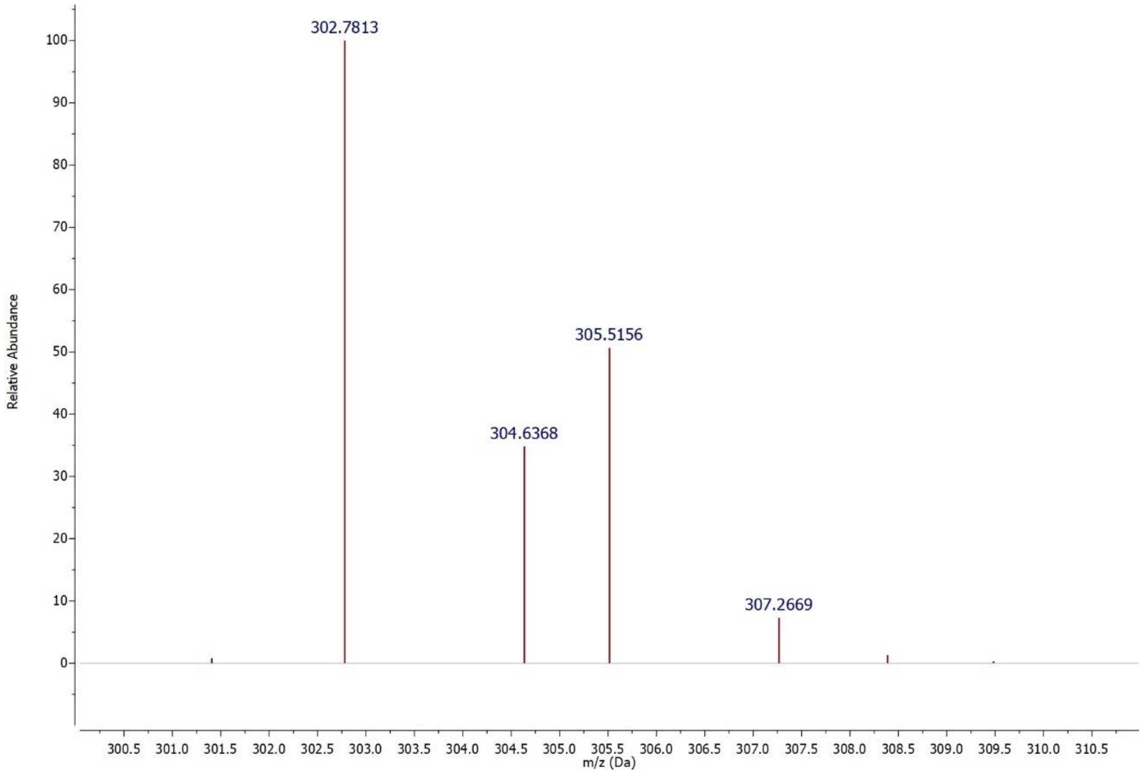

Figure S5. ESI-MS (+) spectrum of 5-[4-((*tert*-butyldimethylsilyl)oxy)phenyl]pyrazin-2-amine (**2a**).

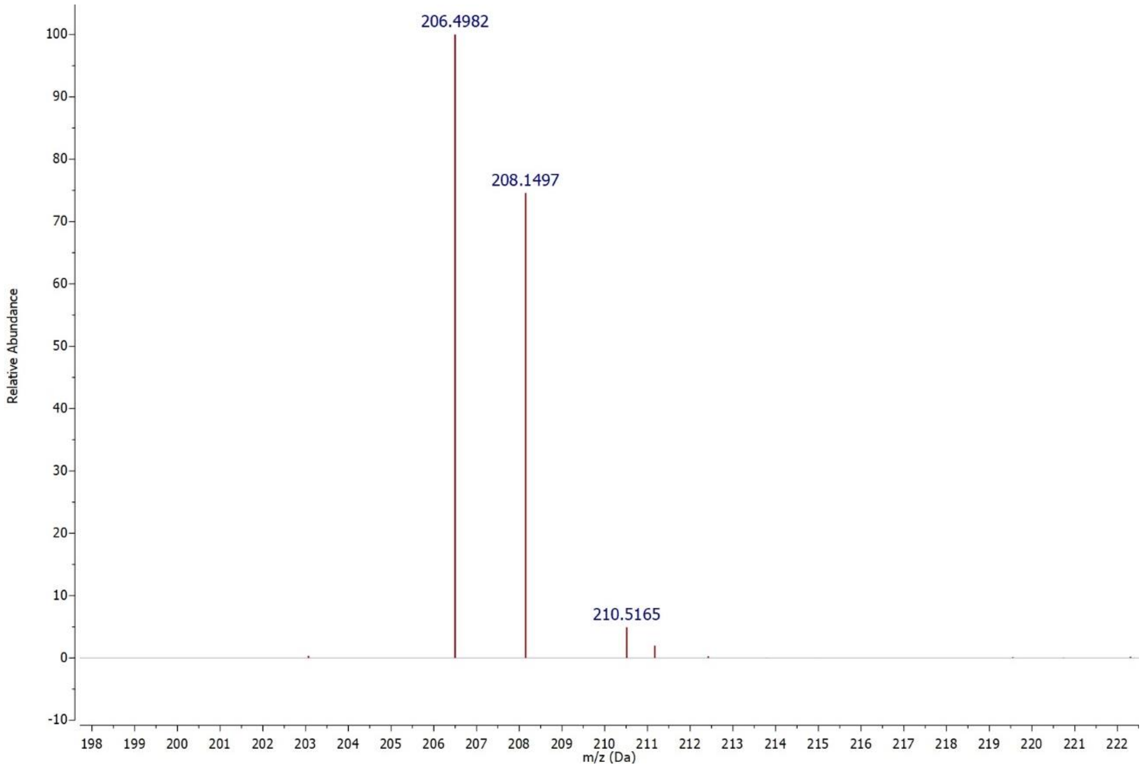

Figure S6. ESI-MS (+) spectrum of 5-(4-chlorophenyl)pyrazin-2-amine (**2b**).

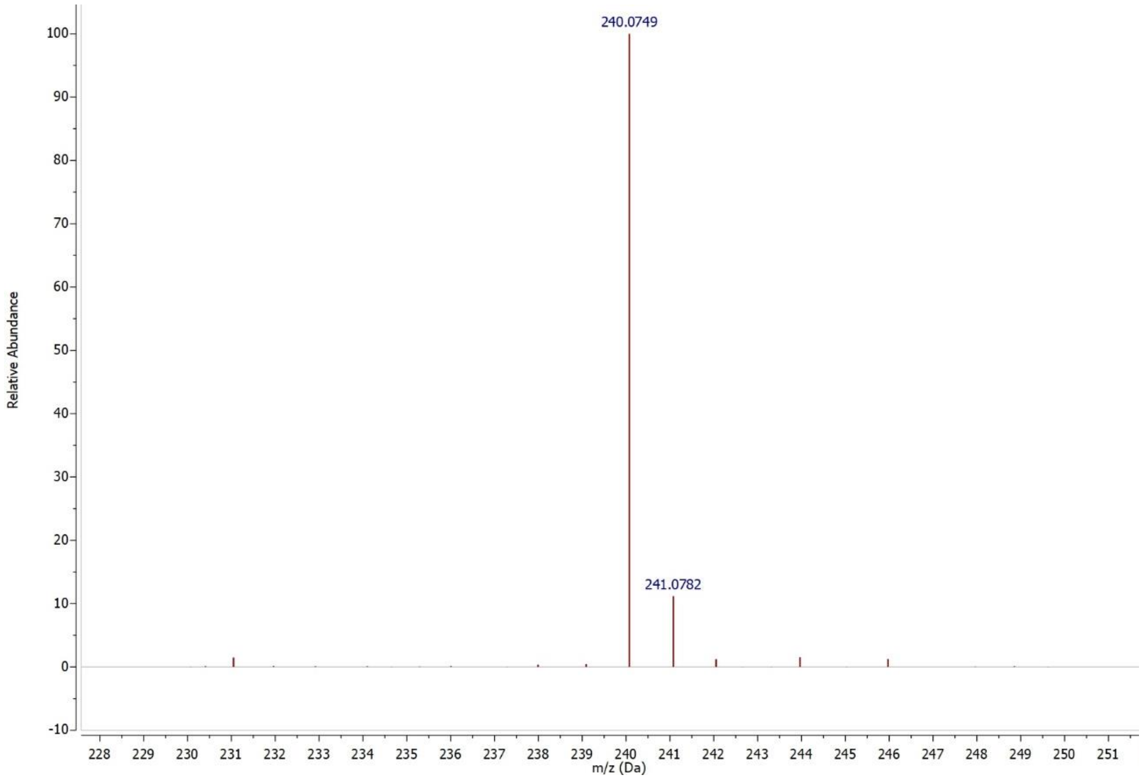

Figure S7. HR-MS ESI (-) spectrum of 6-(4-hydroxyphenyl)-2-methylimidazo[1,2-*a*]pyrazin-3(7*H*)-one (**OH-Cla**).

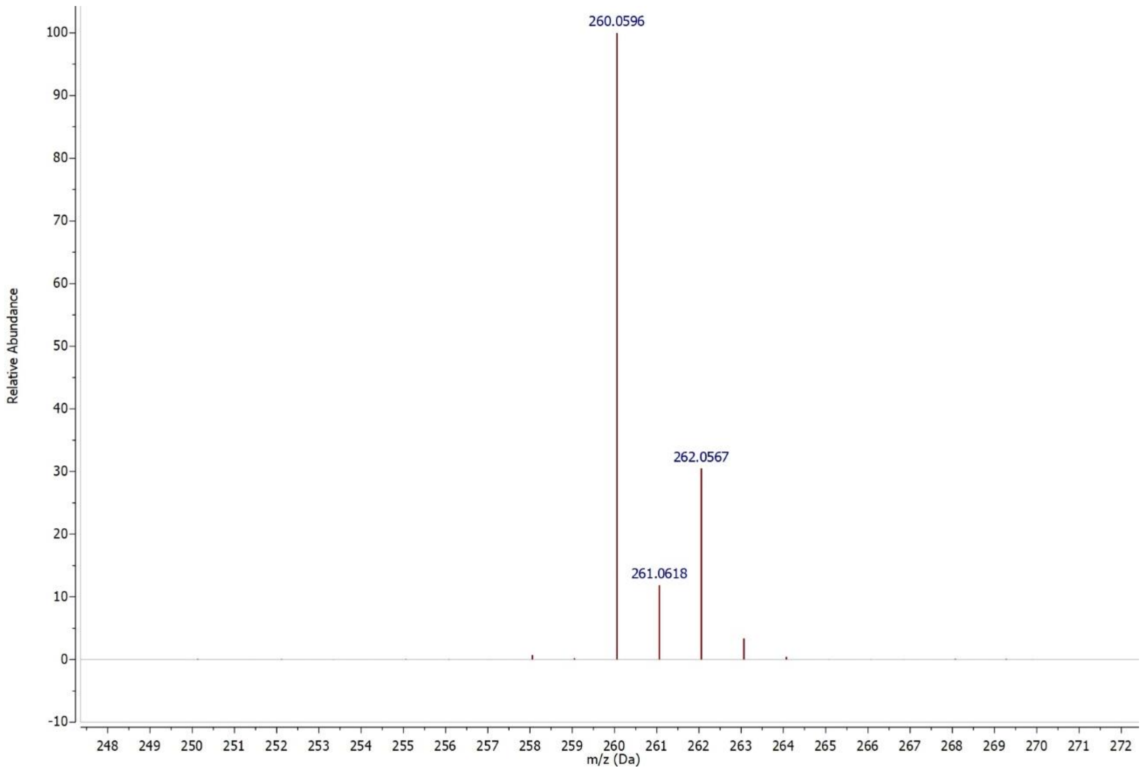

Figure S8. HR-MS ESI (+) spectrum of 6-(4-chlorophenyl)-2-methylimidazo[1,2-*a*]pyrazin-3(7*H*)-one (**Cl-Cla**).

2.3.FT-IR Spectra

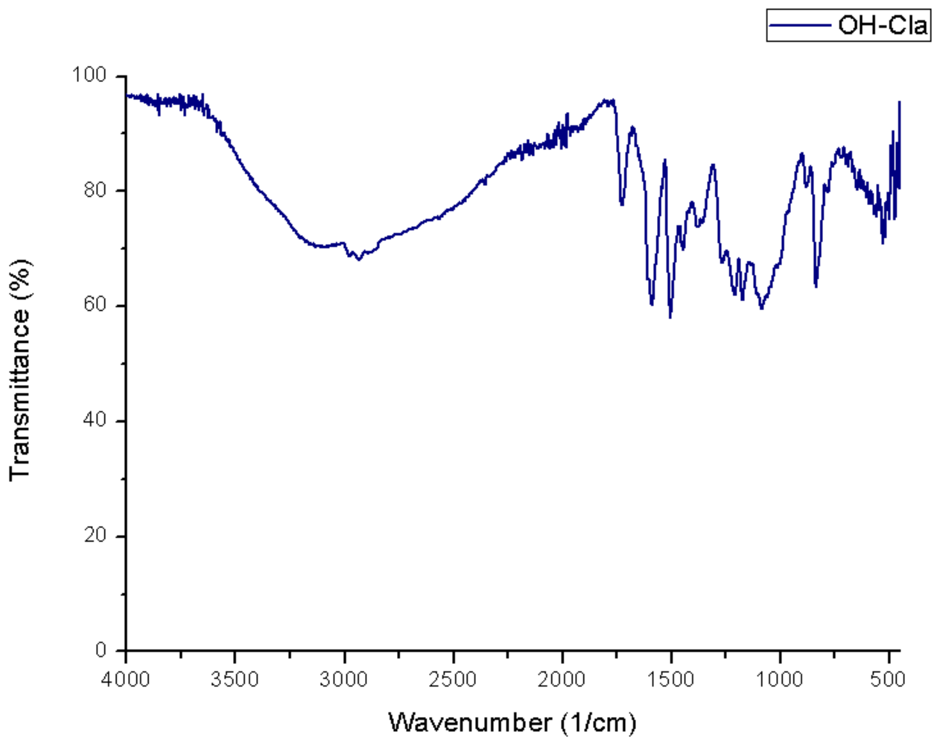

Figure S9. FT-IR spectrum of 6-(4-hydroxyphenyl)-2-methylimidazo[1,2-*a*]pyrazin-3(7*H*)-one (**OH-Cla**).

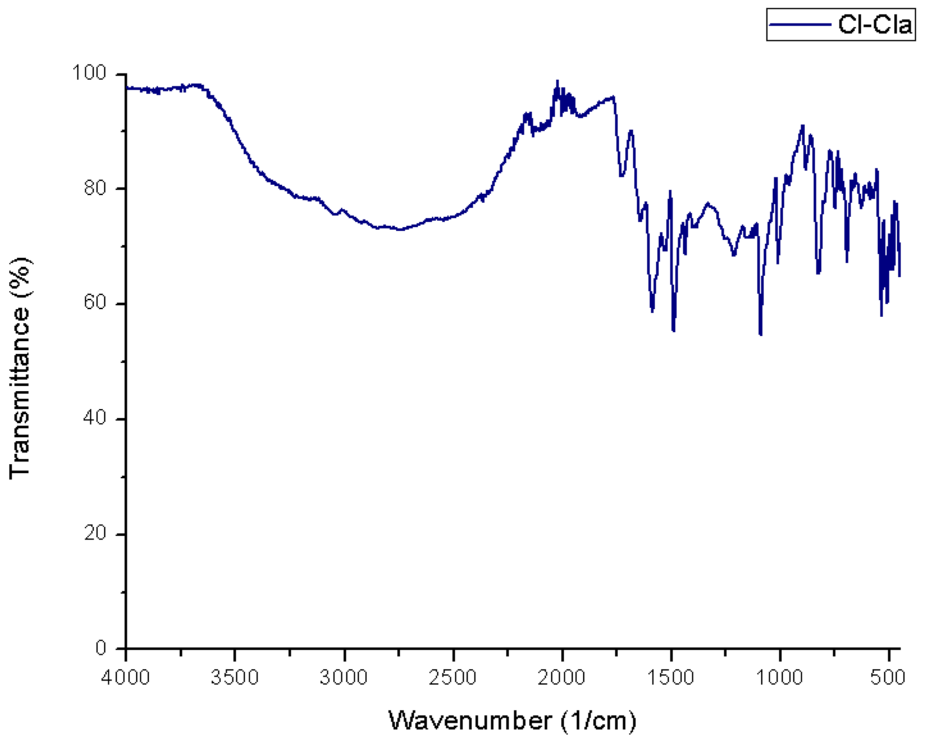

Figure S10. FT-IR spectrum of 6-(4-chlorophenyl)-2-methylimidazo[1,2-*a*]pyrazin-3(7*H*)-one (**Cl-Cla**).

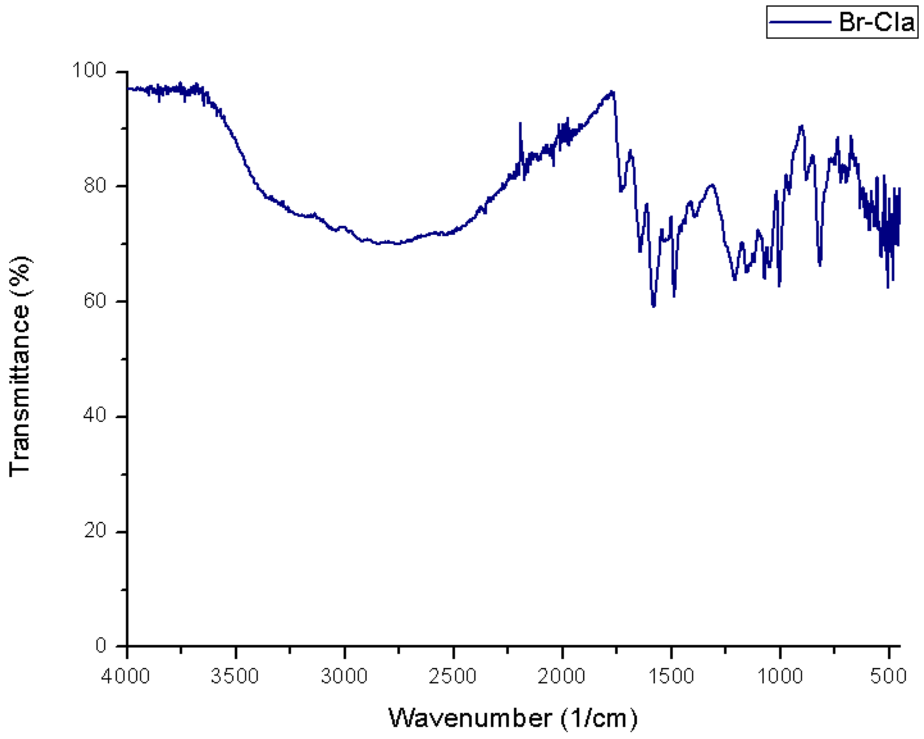

Figure S11. FT-IR spectrum of 6-(4-bromophenyl)-2-methylimidazo[1,2-*a*]pyrazin-3(7*H*)-one (**Br-Cla**).

2.4.HPLC-DAD

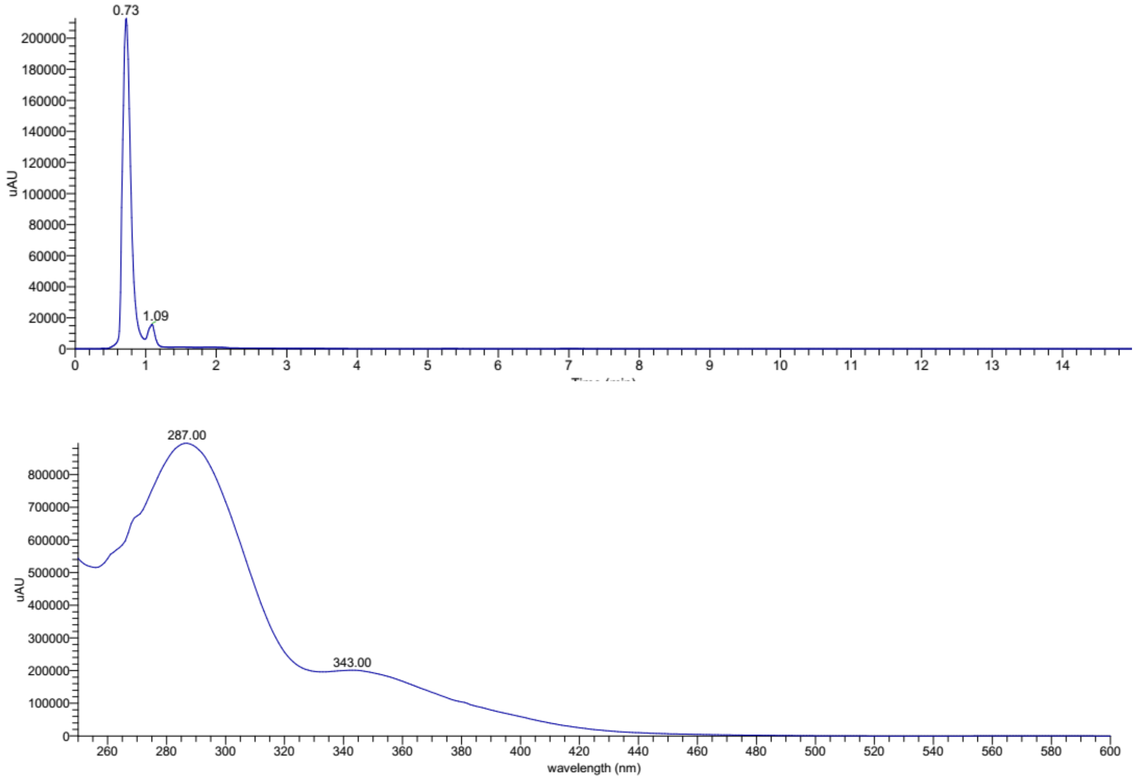

Figure S12. HPLC chromatogram and DAD-UV-Vis spectrum of 6-(4-hydroxyphenyl)-2-methyl-imidazo[1,2-*a*]pyrazin-3(7*H*)-one (**OH-Cla**).

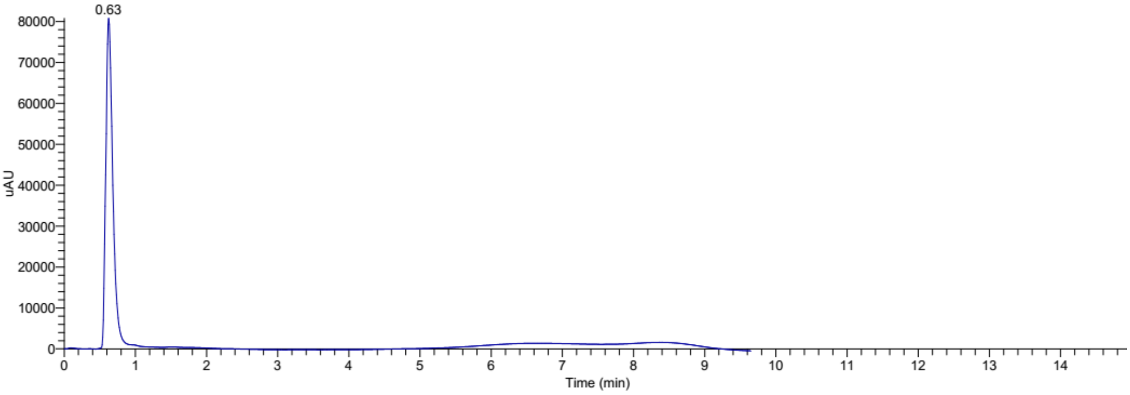

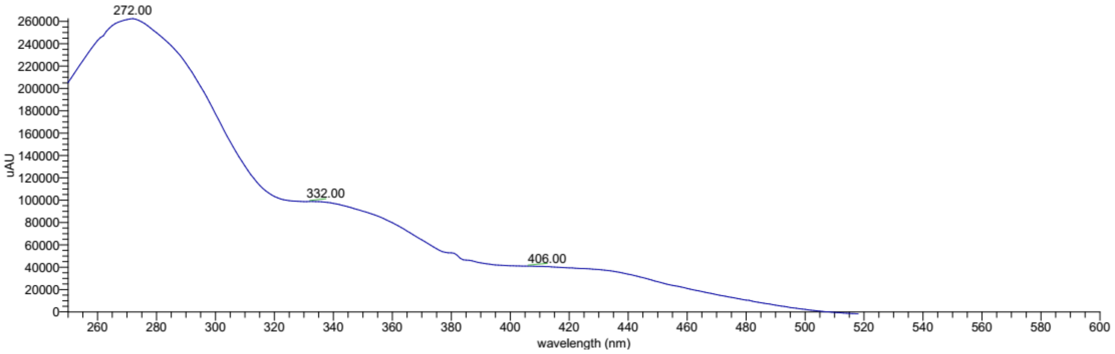

Figure S13. HPLC chromatogram and DAD-UV-Vis spectrum of 6-(4-chlorophenyl)-2-methyl-imidazo[1,2-*a*]pyrazin-3(7*H*)-one (**Cl-Cla**).

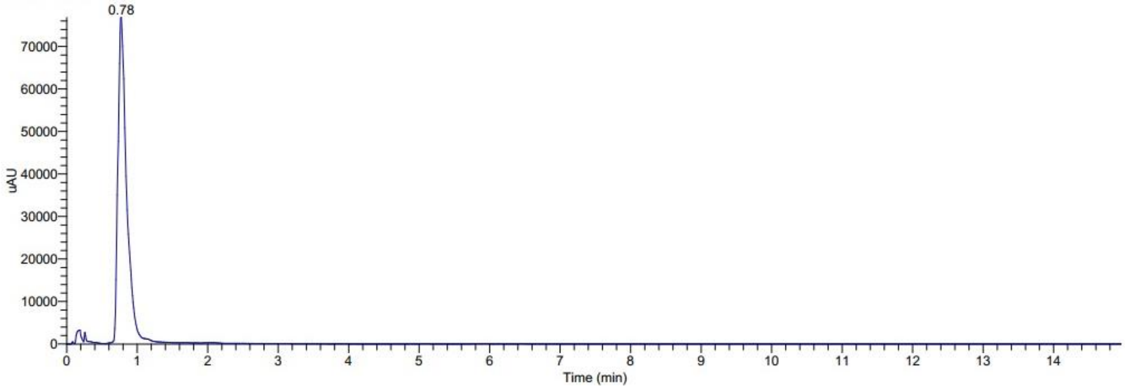

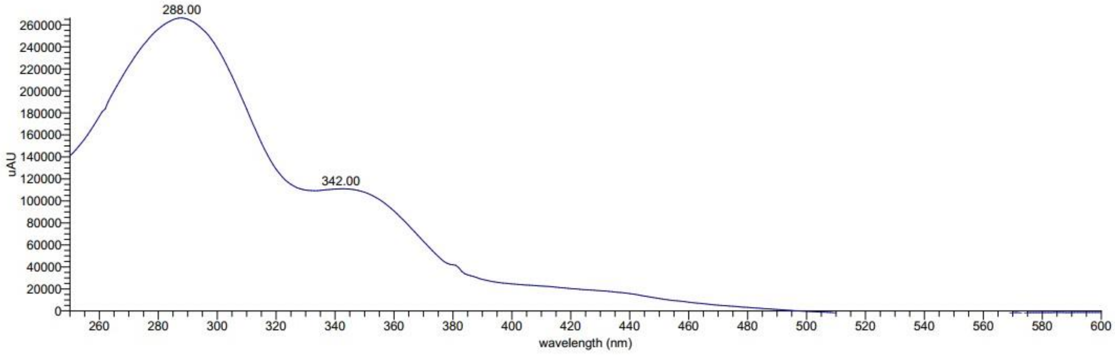

Figure S14. HPLC chromatogram and DAD-UV-Vis spectrum of 6-(4-bromophenyl)-2-methyl-imidazo[1,2-*a*]pyrazin-3(7*H*)-one (**Br-Cla**).

2.5. Absorbance spectra

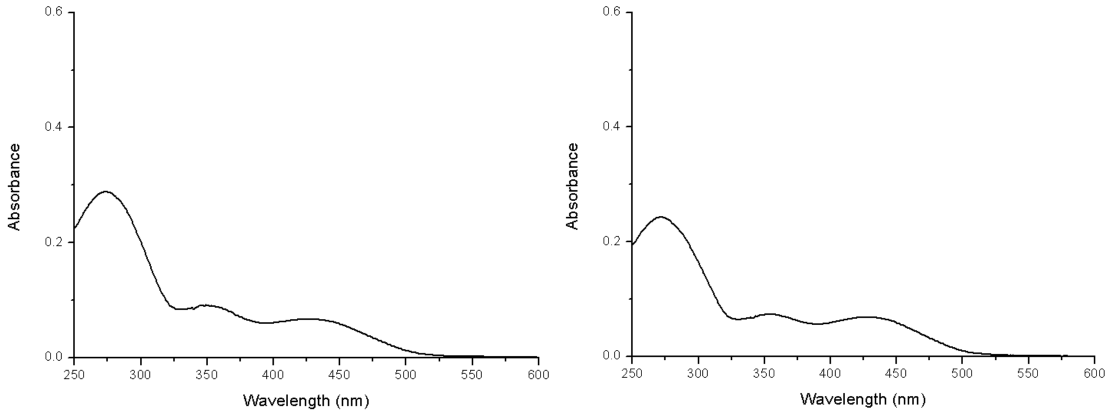

Figure S15. Absorbance spectra of 6-(4-hydroxyphenyl)-2-methyl-imidazo[1,2-*a*]pyrazin-3(7*H*)-one (**OH-Cla**) in MeOH:CH_3CN (1:1) (left), and CH_3CN:water:formic acid (3:2:0.1) (right)

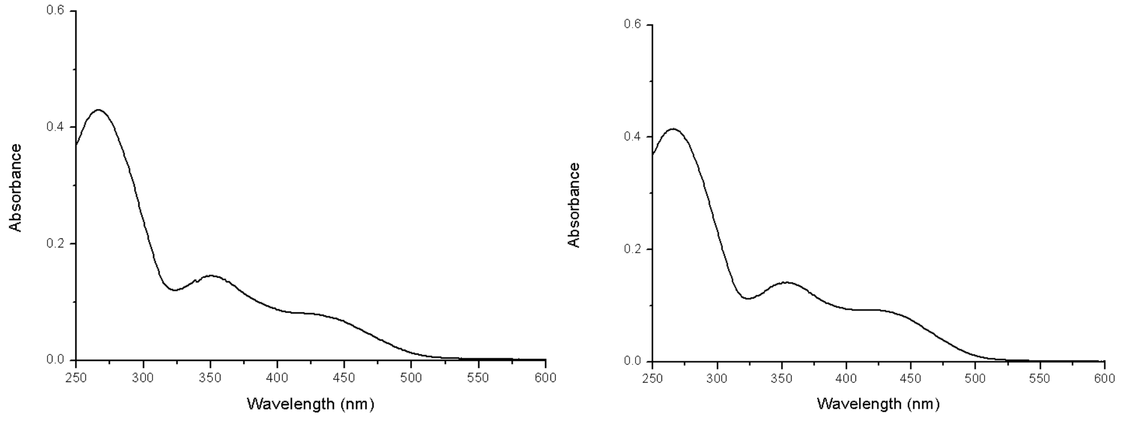

Figure S16. Absorbance spectra of 6-(4-chlorophenyl)-2-methyl-imidazo[1,2-*a*]pyrazin-3(7*H*)-one (**Cl-Cla**) in MeOH:CH_3CN (1:1) (left) and CH_3CN:water:formic acid (3:2:0.1) (right).

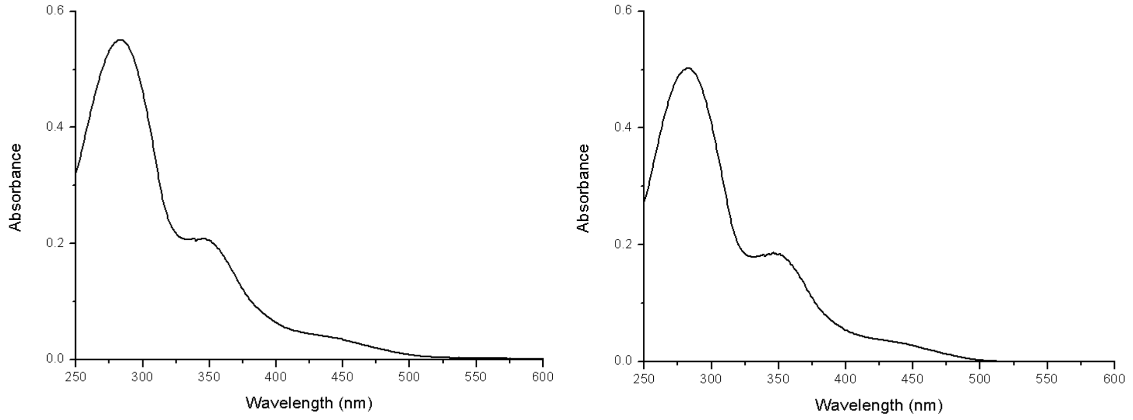

Figure S17. Absorbance spectra of 6-(4-bromophenyl)-2-methyl-imidazo[1,2-*a*]pyrazin-3(7*H*)-one (**Br-Cla**) in MeOH:CH_3CN (1:1) (left) and CH_3CN:water:formic acid (3:2:0.1) (right).

2.6.Photophysical characterization

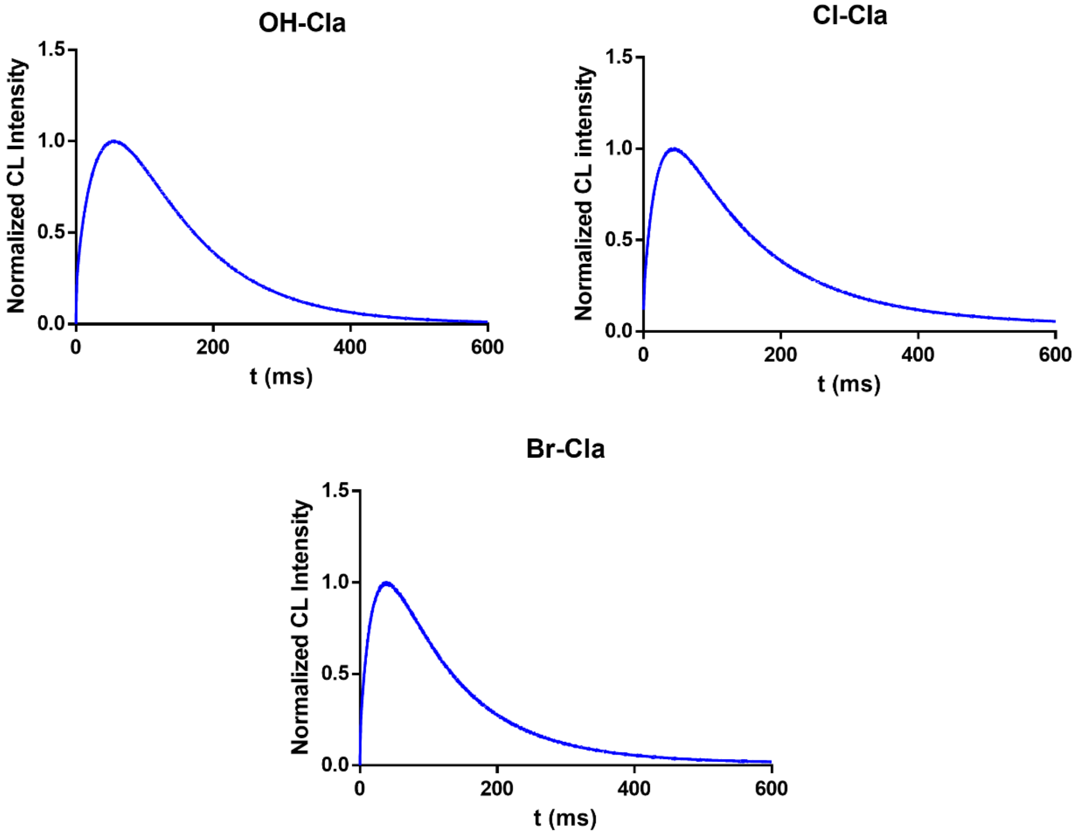

Figure S18. Normalized chemiluminescence profiles of **-OH**, **-Cl**, and **Br-Cla** in DMF-acetate buffer pH 5.14 (0.68%).

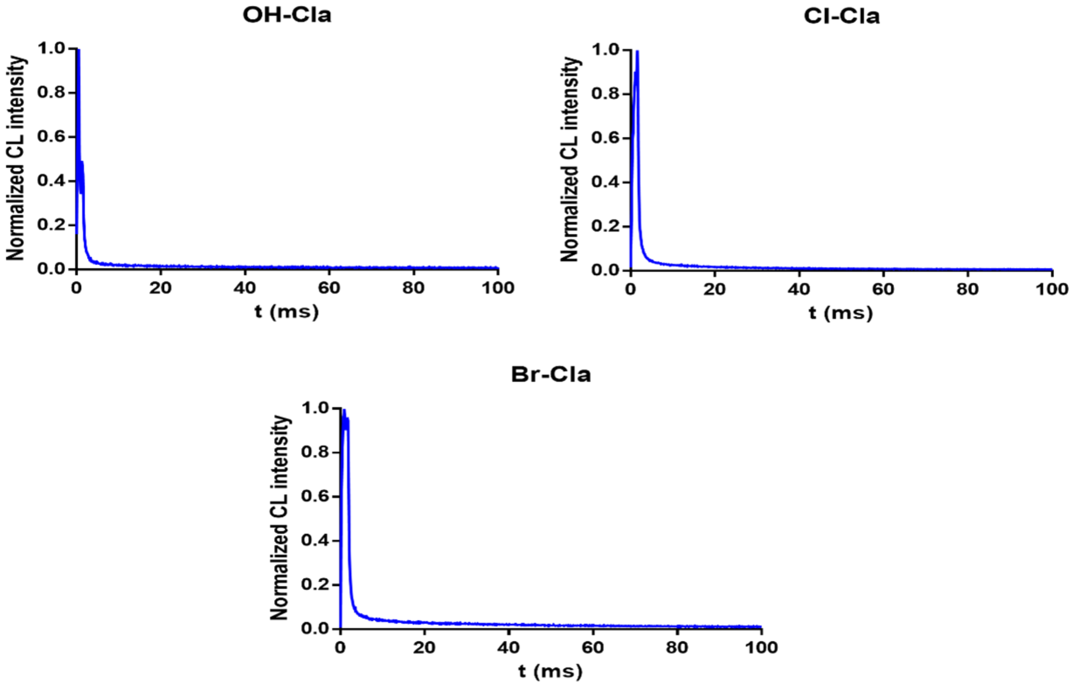

Figure S19. Normalized chemiluminescence profiles of **-OH**, **-Cl**, and **Br-Cla** in the presence of 15 mg of KO_2 in methanol.

2.7. Cellular Assays

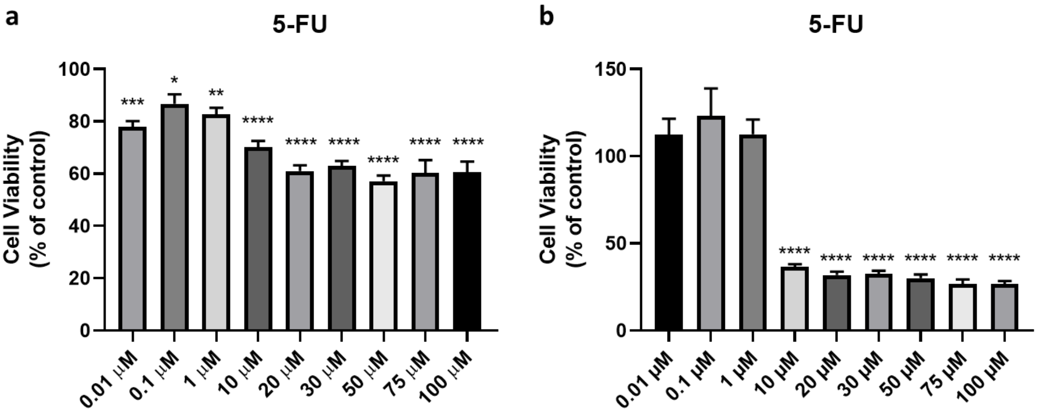

Figure S20. Cellular viability assays of HT-29 (**a**) and SH-SY5Y (**b**) cells after 48 hours of incubation with **5-FU**. * Statistically significant vs. control at $p < 0.05$. ** statistically significant vs. control at $p < 0.01$ *** statistically significant vs. control at $p < 0.001$ **** statistically significant vs. control at $p < 0.0001$.

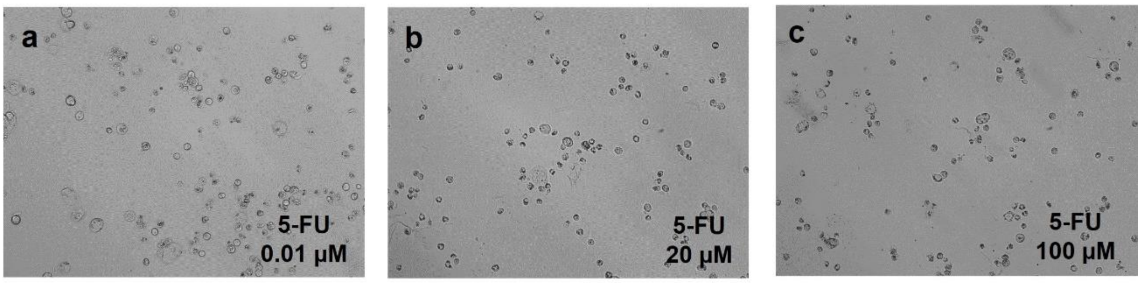

Figure S21. Microscopic cellular visualization of HT-29 cells after 48 hours of incubation with **5-FU** at (**a**) 0.01 µM, (**b**) 20 µM and (**c**) 100 µM.

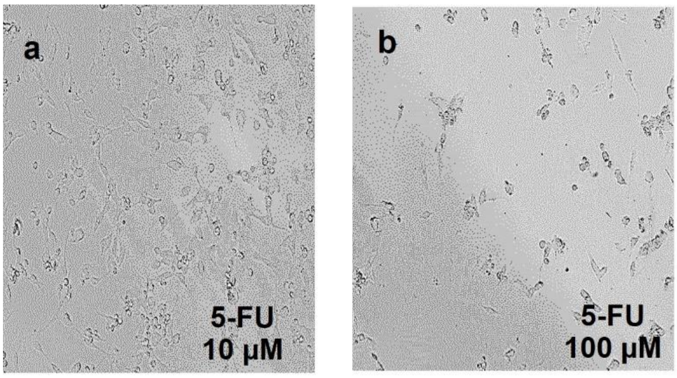

Figure S22. Microscopic cellular visualization of SH-SY5Y cells after 48 hours of incubation with with **5-FU** at (**a**) 10 µM and (**b**) 100 µM.
